# Scalable Li‐Ion Battery with Metal/Metal Oxide Sulfur Cathode and Lithiated Silicon Oxide/Carbon Anode

**DOI:** 10.1002/cssc.202400615

**Published:** 2024-09-24

**Authors:** Edoardo Barcaro, Vittorio Marangon, Dominic Bresser, Jusef Hassoun

**Affiliations:** ^1^ Department of Chemical, Pharmaceutical and Agricultural Sciences University of Ferrara via Fossato di Mortara 17 44121 Ferrara Italy; ^2^ Helmholtz Institute Ulm (HIU) Helmholtzstrasse 11 89081 Ulm Germany; ^3^ Karlsruhe Institute of Technology (KIT) P.O. Box 3640 76021 Karlsruhe Germany; ^4^ Graphene Labs Istituto Italiano di Tecnologia via Morego 30 16163 Genova Italy

**Keywords:** Li−S battery, Li-ion full-cell, MnO_2_/Sn, SiO_2_, Graphene

## Abstract

A Li‐ion battery combines a cathode benefitting from Sn and MnO_*2*_ with high sulfur content, and a lithiated anode including fumed silica, few layer graphene (FLG) and amorphous carbon. This battery is considered a scalable version of the system based on lithium‐sulfur (Li−S) conversion, since it exploits at the anode the Li‐ion electrochemistry instead of Li‐metal stripping/deposition. Sn and MnO_2_ are used as cathode additives to improve the electrochemical process, increase sulfur utilization, while mitigating the polysulfides loss typical of Li−S devices. The cathode demonstrates in half‐cell a maximum capacity of ~1170 mAh g_S_^−1^, rate performance extended over 1 C, and retention of 250 cycles. The anode undergoes Li‐(de)alloying with silicon, Li‐(de)insertion into amorphous carbon, and Li‐(de)intercalation through FLG, with capacity of 500 mAh g^−1^ in half‐cell, completely retained over 400 cycles. The full‐cells are assembled by combining a sulfur cathode with active material loading up to 3 mg cm^−2^ and lithiated version of the anode, achieved either using an electrochemical pathway or a chemical one. The cells deliver at C/5 initial capacity higher than 1000 mAh g_S_^−1^, retained for over ~40 % upon 400 cycles. The battery is considered a promising energy storage system for possible scaling‐up in pouch or cylindrical cells.

## Graphical abstract

Scalable Li‐ion sulfur cell combines composite cathode added with Sn and MnO_2_, and pre‐lithiated silicon oxide/carbon anode. The Li_y_SiO_x_‐CM|S‐SM full‐cell enables at 1.8 V the multi‐step sulfur conversion and the anode (de)alloying/(de)insertion over 400 cycles, with initial capacity exceeding 1000 mAh g_S_^−1^, and retention depending on the cell setup.

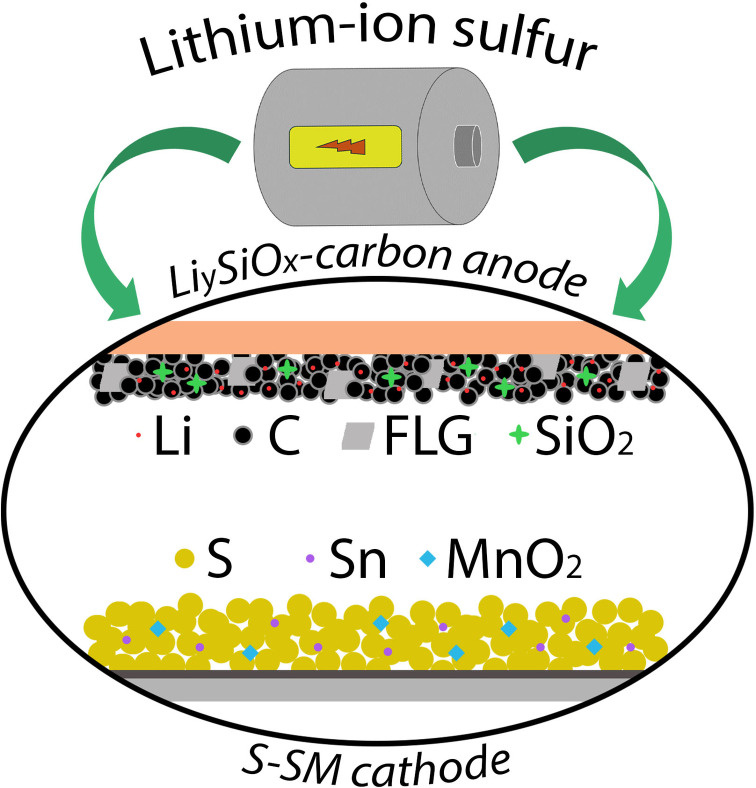

## Introduction

### The Sulfur Cathode

The demand for Li‐ion batteries (LIBs) is expected to rise relentlessly due to their applications as fundamental power sources in a vast range of technologies, ranging from portable electronics to electric vehicles, and as essential stationary energy storage systems. The latter application is foreseen considering that the installed grid‐scale battery storage capacity, which stood to ~28 GW at the end of 2022, is expected to expand to 35‐fold within 2030 to nearly 970 GW in the Net Zero Scenario.^[1]^ On the other hand, materials availability and cost have been identified as key factors for triggering a sustainable development with controlled economic, environmental and geopolitical impact.^[2]^ Therefore, suitable alternatives to common LIBs with increased energy density compared to the typical 150–300 Wh kg^−1^ range, and with electrodes based on abundant, easily accessible, and highly performing materials are needed to actually promote a step forward.[^3^, ^4^, ^5^, ^6^] Elemental sulfur (S_8_) can electrochemically react with lithium delivering a theoretical capacity of 1675 mAh g_S_^−1^, according to the reversible redox reactions S_8_+16Li^+^+16e^−^ ⇆ 8Li_2_S.^[7]^ However, energy storage systems based on Li−S electrochemistry may be particularly entangled, since it involves the formation of various intermediate anions and radicals, depending on the nature and physical state (solid or liquid) of the electrolyte, salt concentration, electrode morphology, and active material content.[^8^, ^9^, ^10^] Indeed, S_8_ is almost an insulator and can undergo a series of compositional and structural rearrangements in the Li−S system leading to the formation of soluble polysulfides (Li_2_S_x_ with 4≤×≤8) with significant changes in the cathode morphology, thus posing great practical challenges and drawbacks such as modest rate capability, rapid capacity fading, low material utilization, and poor mechanical stability.[^8^, ^11^, ^12^] Furthermore, the full‐discharge products of Li−S cell (i. e., Li_2_S_2_ or Li_2_S) can precipitate from the electrode as a solid limiting the reversibility of the electrochemical process and the delivered capacity.^[13]^ Therefore, suitable current collectors, conductive matrixes, and additives in the electrodes and/or the electrolytes are requested to boost the cell performances, and ensure appropriate power and energy density.[^14^, ^15^, ^16^, ^17^] Another limiting issue of the Li−S system is represented by the Li‐metal anode which can degrade the electrolyte unless a stable solid‐electrolyte‐interphase (SEI) is formed, thus hampering the long‐term cycling of the battery.[^18^, ^19^] Soluble polysulfides can also diffuse from the cathode to the Li anode to directly react and precipitate, or can travel back to the cathode to be newly oxidized through a shuttle reaction without any charge accumulation.^[13]^ This undesired process can typically lead to material loss, promote dendrite formation, and limit both material utilization and cell efficiency.^[20]^

### The Silicon Oxide‐Carbon Anode

Despite the recent improvements, the use of a Li‐metal anode may still represent a potential safety issue that could limit the actual use of Li−S high‐energy storage devices, which are presently affected by short‐to‐medium term cycling issues. Further improvement of the SEI characteristics and safety content can be achieved by tuning the electrolyte nature and composition.[^17^, ^21^, ^22^, ^23^, ^24^] A valid approach to fully exploit the Li‐metal capacity (i. e., 3860 mAh g^−1^), while keeping a negative‐to‐positive ratio (N/P) ≤ 3, has been represented by the use of solid‐state electrolytes.^[10]^ On the other hand, the replacement of this challenging metal with alternative Li‐ion anodes to get a configuration similar to the *rocking‐chair* battery based on insertion or intercalation materials appeared a suitable strategy to promote the cell cycle life, efficiency and safety.[^25^, ^26^] Among the various anodes proposed for this matter, carbons with various morphologies, and Li−Si alloying composites have revealed the most suitable performance in terms of high delivered capacity sufficient to match the one originating from Li−S conversion process, and adequate cycle life.[^27^, ^28^] However, the use of an alternative anode in a Li−S system requires a lithiated version of the electrode itself to ensure a *lithium reservoir* for allowing the reversible operation, and avoid the cell decay or even failure.^[29]^ The lithiation process has been initially proposed by adopting an electrochemical route, according to which the material is cycled in a separated half‐cell as the working electrode until achieving the discharged (lithiated) state, and subsequently retrieved for the use in full‐cell,[^30^, ^31^] while additional routes involved the implementation of sacrificial additives.^[32]^ An alternative strategy foreseen the chemical pre‐treatment by bringing the electrode in close contact with an electrolyte‐wetted metallic lithium foil for a determined time (e. g., capillary contact), in order to favor the lithiation prior to full‐cell assembly.[^30^, ^31^] Despite the former pathway may ensure a more controlled lithiation of the anode for proper operation in Li‐ion sulfur cell, the latter one is considered a more scalable process for achieving electrode films for direct application in pouch, cylindrical or prismatic cell. Therefore, the scaling‐up of this battery may be actually achieved by setting up a suitable electrolyte‐to‐sulfur (E/S) ratio, and by tuning the sulfur cathode and the anode amounts into a Li‐ion configuration with an adequate N/P ratio, defined as the ratio between the capacity delivered in half‐cell by charging the anode (i. e., de‐lithiation of the negative electrode in full‐cell) and discharging the cathode (i. e., lithiation of the positive electrode in full‐cell).^[23]^

### Aim of the Work

Herein, we propose a Li‐ion sulfur cell with enhanced cathode and anode materials into a rational combination ensuring, at the same time, high‐capacity, long cycle life and possible scalability. The sulfur cathode is characterized by a reinforced polysulfide control achieved by including MnO_2_, and a rate capability favored by nanometric Sn particles, whilst the electrode conductivity and thin configuration are ensured by including FLG in the cathodic slurry, and multiwalled carbon‐nanotubes (MWCNTs) in the current collector.[^14^, ^15^, ^16^] The Sn nanoparticles are introduced to act as the conductive framework in order to replace carbon additives, which should be employed by relevant amount for ensuring good conductivity. In addition, enhanced carbons usually require complex synthesis procedures, and present a high specific volume that may affect the overall energy density. Instead, metal nanoparticles can be tuned with a limited concentration thanks to their outstanding conducting character as previously demonstrated.[^14^, ^33^] In addition, both Sn and MnO_2_ are abundant and non‐toxic materials, thus representing a rational choice for combination with the environmentally friendly sulfur to ensure remarkable conductivity and lithium polysulfide retention, respectively, even with concentration limited to 5 wt % each in the sulfur‐based cathode composite. Thus, the scalability of the new S:Sn:MnO_2_ composite, indicated as S‐SM in the text, resides in a simple synthesis which foresees mixing at mild temperature S, Sn and MnO_2_, thus avoiding chemical reactions or the use of solvents, and relies on mechanical grinding/milling to achieve the final powder. The above process is actually accepted as scalable step for large‐scale production of lithium‐ion battery electrodes.^[34]^ Therefore, the S‐SM composite is initially investigated in terms of structure, morphology, thermal behavior, and electrochemical proprieties in lithium half‐cell. Remarkably, the increase of the sulfur loading from 2 to ~6 mg cm^−2^ and the concomitant decrease of the E/S ratio from 10 to 5 μL mg^−1^ only partially affect the half‐cell performance. Instead, the composite anode has been synthesized in our previous work using amorphous carbon derived from sucrose, FLG, and fumed silica.^[35]^ The anode composite material, indicated as SiO_x_‐CM, has been previously characterized for application in a Li‐ion battery using a conventional electrolyte and an intercalation cathode.^[35]^ In this work, we provide chemical and electrochemical pre‐lithiation of the material to achieve a Li_y_SiO_x_‐CM phase, suitable for application in full‐cell in combination with the S‐SM cathode. Prior to application, the pristine anode and its lithiated version are studied in half‐cells using the electrolyte typically employed in the Li−S system formed by 1,3‐dioxolane (DOL) and 1,2‐dimethoxyethane (DME) solvents, lithium bis(trifluoromethanesulfonyl)imide (LiTFSI) conducting salt, and LiNO_3_ passivating additive.^[13]^ The full‐cells are subsequently assembled with sulfur loading exceeding 1 mg cm^−2^, an E/S ratio of 15 μL mg^−1^, and a N/P ratio below 1.2. The cells revealed remarkable cycle life, high efficiency, and moderate retention, even when tested in challenging conditions exploiting a sulfur loading approaching 3 mg cm^−2^ and E/S ratio limited to 8 μL mg^−1^. We believe that the results reported herein may boost the development of full‐cells to achieve prototypes of practical interest, upon further optimization of sulfur loading as well as of E/S and N/P ratios.[^23^, ^36^]

## Results and Discussion

**Figure 1:**
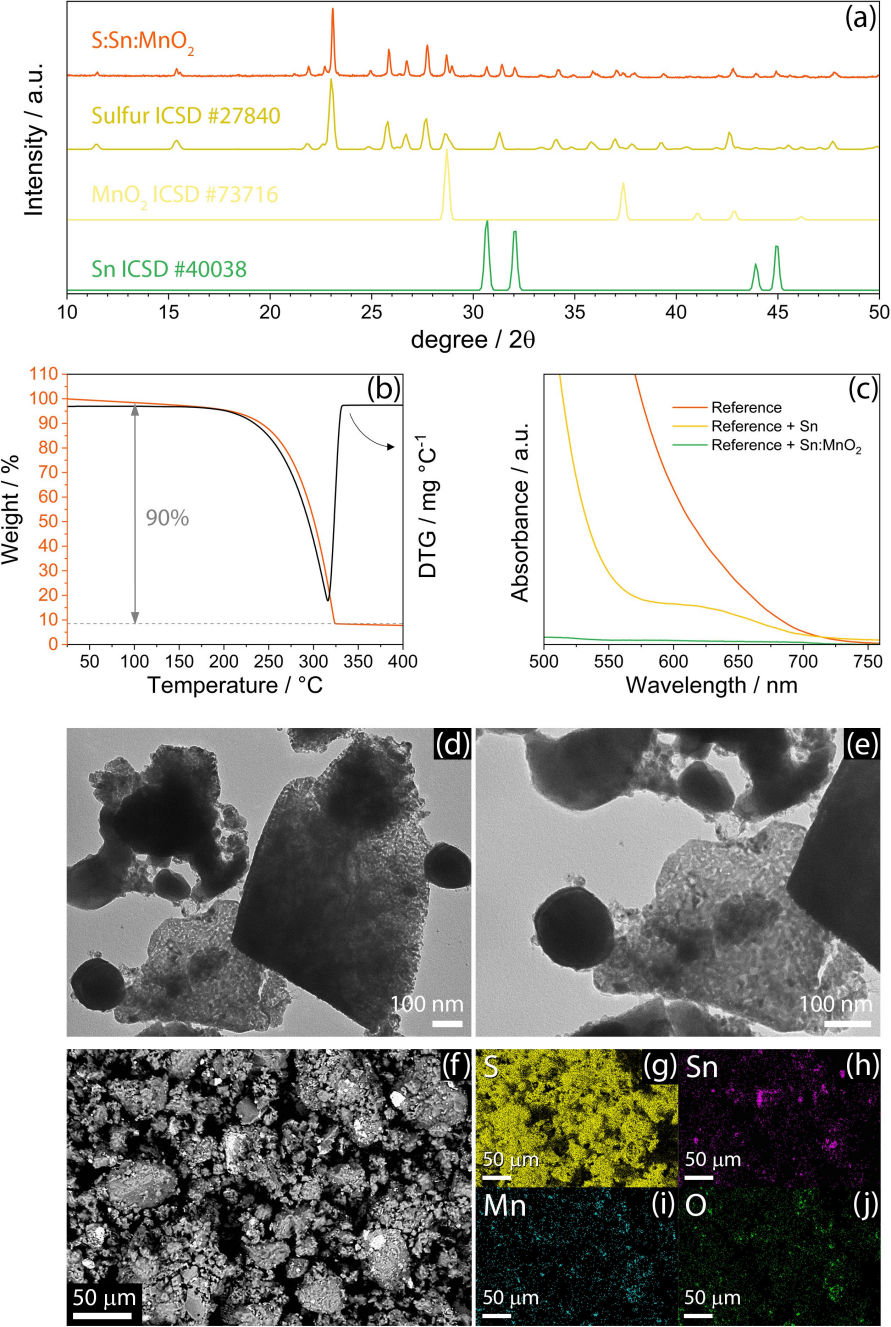
Physical‐chemical characterization of the S‐SM powder. (**a**) X‐ray diffractograms for the S‐SM sample (orange). Reference data for sulfur (S_8_, ICSD#27840, dark yellow), manganese dioxide (β‐MnO_2_, ICSD#73716, light yellow), and tin (β‐Sn, ICSD#40038, green), are reported for comparison. (**b**) TGA performed under dry N_2_ flow with heating rate of 5 °C min^−1^ from 25 to 400 °C, and corresponding DTG. (**c**) UV‐vis measurements performed on DOL:DME, 1 mol kg^−1^ LiTFSI, 1 mol kg^−1^ LiNO_3_, 0.5 wt % Li_2_S_8_ solutions displayed in Figure S1 (Supporting Information) in the 500–750 nm wavelength range, without any powder addition (reference, red), with addition of Sn (yellow) or Sn:MnO_2_ 1 : 1 *w/w* (green). (**d** and **e**) TEM images of the S‐SM powder at various magnifications. (**f**) SEM image of S‐SM powder acquired in backscattered electrons mode, and corresponding EDS elemental maps of (**g**) sulfur (yellow), (**h**) tin (purple), (**i**) manganese (cyan), and (**j**) oxygen (green).

Structure, morphology and composition of the S‐SM material are depicted in Figure 1. The X‐ray diffraction (XRD) pattern between 10° and 50° of 2*θ* in Figure 1a reveals the exclusive presence of orthorhombic sulfur (S_8_, ICSD#27840)_,_ manganese dioxide (β‐MnO_2_, ICSD#73716), and tin (β‐Sn, ICSD#40038) without any additional reflection. This response likely excludes the formation of crystalline by‐products or impurities, which is mainly due to the mild temperature of 125 °C used for the synthesis (see Experimental section for details).^[37]^ The actual amount of sulfur in the S‐SM material is detected through thermogravimetric analysis (TGA) under N_2_ and the resulting curve is reported in Figure 1b. The figure exhibits a sulfur content as high as 90 % into the composite, which is expected to facilitate the tuning of the active material loading in the electrodes for increasing the energy density of the cells. Furthermore, this synthetic pathway may be reasonably scaled‐up to achieve practical production, since it involves mild heating and mechanical milling/grinding in a solvent‐free environment. The differential curve (DTG, Figure 1b) associated with the TGA analysis evidences a weight loss related to sulfur evaporation evolving through a single peak centered at 315 °C.^[38]^ It is worth noting that residual weight corresponds to Sn and MnO_2_ fractions which are inert under the experimental constrains. The ability of Sn and MnO_2_ in retaining lithium polysulfides (Li‐PS) is qualitatively evaluated by UV‐vis measurements in Figure 1c. Vials containing DOL and DME dissolving LiTFSI, LiNO_3_, and 0.5 wt % of Li_2_S_8_ polysulfide are added either with Sn or Sn:MnO_2_ 1 : 1 *w/w* (see further details in Experimental section). All the solutions photographed in Figure S1 (Supporting Information) initially exhibit the same dark‐red color of the reference one (left‐hand side) due to the dissolved Li_2_S_8_, in spite of the presence of Sn (central position) or Sn:MnO_2_ 1 : 1 *w/w* (right‐hand side). This intense color is strongly mitigated upon 60 minutes of contact with Sn:MnO_2_ mixture (Figure S1b, right‐hand side), slightly changed in contact with Sn powder within the same time interval (Figure S1b, central position), whilst the reference solution remains obviously unaltered (Figure S1b, left‐hand side). The color attenuation is mainly ascribed to the relevant Li‐PS retention ability of the transition metal oxide rather than the metallic tin, which is instead included in the sulfur composite principally to enhance the conductivity. The better polysulfide retention ability of MnO_2_ is confirmed by UV‐vis measurements performed on reference Li_2_S_8_ solution, and on solutions held in contact with Sn and Sn:MnO_2_ 1 : 1 *w/w* (Figure 1c). Indeed, the analysis shows the characteristic signal of Li‐PS species in the visible region between 750 and 500 nm with a relevant intensity for the reference solution, while an attenuated signal is observed for the one held in contact with nanometric Sn, which almost vanishes for the solution aged with Sn:MnO_2_ 1 : 1 *w/w*. The Li‐PS retention ability may be influenced by the intrinsic interactions between Sn and MnO_2_ and Li_2_S_x_ species, as well as by the morphology of the sample. Hence, the electron microscopy in Figure 1d–j enables to investigate the morphological features of S‐SM powder. The transmission electron microscopy (TEM) images reported in Figure 1d and e show substantial differences between primary micrometric and nanometric domains. The amorphous or defined flake‐like particles with micron or sub‐micron size can be attributed either to S or MnO_2_. Instead, the nanometric domains certainly identify regular Sn spherules smaller than 200 nm as observed in previous work (see Figure S2 in Supporting Information for further image).^[14]^ It is worth mentioning that the nanometric size observed for the Sn particles is particularly suggested for possibly increasing the electron conductivity by shortening the ion‐diffusion path, thus improving the kinetics of the Li−S electrochemical processes.^[14]^ In addition, the scanning electron microscopy (SEM) image (Figure 1f) indicates that the S‐SM particles are aggregated in the composite to form clusters with size ranging from 1 μm or smaller to about few dozen μm. These macroscopic aggregates can avoid possible electrolyte degradation, which may be instead promoted by dispersed nanometric particles.^[15]^ Furthermore, the X‐ray energy dispersive spectroscopy (EDS) performed on the above SEM (Figure 1g–j) indicates a well uniform distribution of sulfur (Figure 1g) and MnO_2_ (Figure 1i and j), while Sn exhibits the dispersion of isolated nanometric particles alternated with micrometric clusters in the active material matrix.

**Figure 2:**
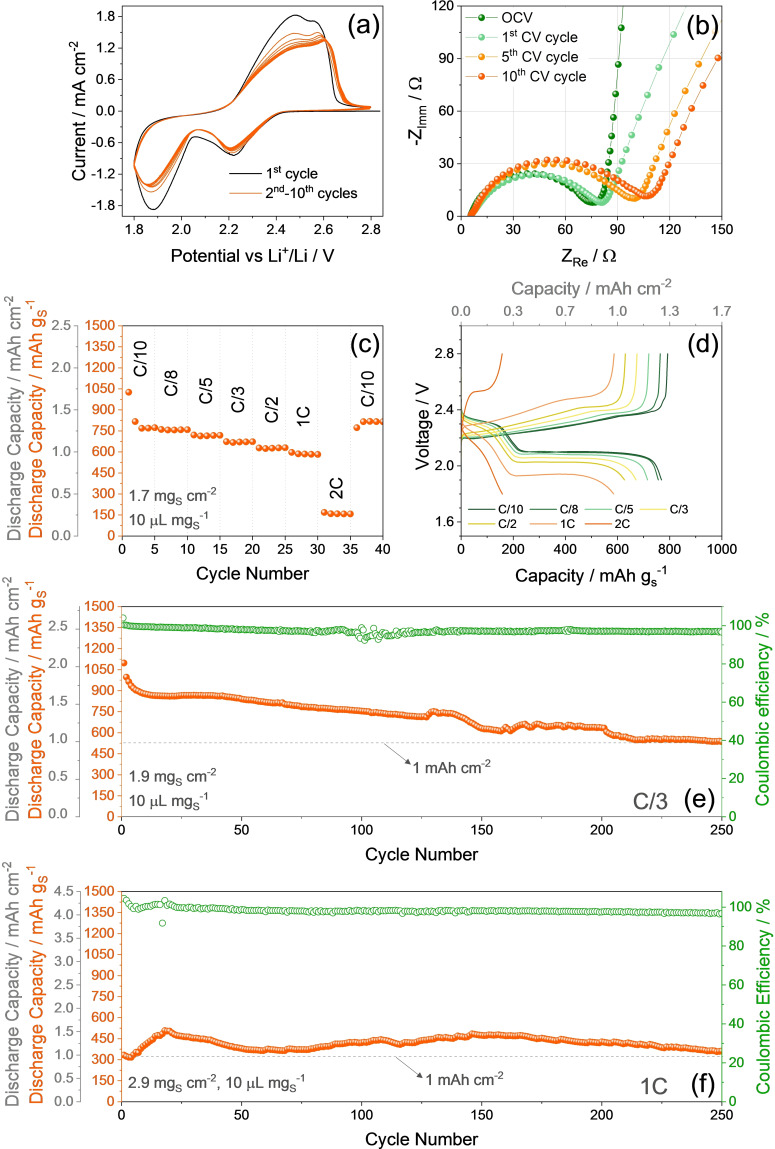
Electrochemical characterization of the S‐SM material in lithium half‐cells using the DOL:DME, 1 mol kg^−1^ LiTFSI, 1 mol kg^−1^ LiNO_3_ electrolyte. (**a**) CV profiles, (**b**) Nyquist plot recorded by EIS. CV potential range: 1.8–2.8 V *vs*. Li^+^/Li, scan rate 0.1 mV s^−1^. EIS performed at the OCV cell condition and upon CV after the 1^st^, 5^th^, and 10^th^ cycle between 500 kHz and 100 mHz; voltage signal: 10 mV. All CV and EIS performed at 25 °C. (**c** and **d**) Rate capability test at increasing current rate of C/10, C/8, C/5, C/3, C/2, 1 C, and 2 C, before lowering back to C/10 after 35 cycles (1 C=1675 mA g_S_^−1^), in the 1.7–2.8 V (for 1 C and 2 C) and 1.8–2.8 V (for lower C‐rates) voltage ranges, in terms of (**c**) discharge capacity trend *vs*. cycle number (additional left *y*‐axis exhibits areal capacity), and (**d**) voltage profiles of the 3^rd^ cycle for each current rate (top *x*‐axis shows areal capacity). (**e** and **f**) Cycling trend of galvanostatic measurements performed at (**e**) C/3 with voltage range of 1.7–2.8 V, and (**f**) 1 C at voltage range of 1.6–2.8 V (right *y*‐axes show CE, additional left *y*‐axes exhibit areal capacity). See corresponding voltage profiles in Figure S3 (Supporting Information). All galvanostatic tests are performed at 30 °C.

Prior to application in full‐cell, the S‐SM composite is electrochemically investigated in lithium half‐cell and the results are reported in Figure 2. The cyclic voltammetry (CV) in Figure 2a displays during the first cycle a reversible conversion process, evolving by two reduction peaks at about 2.2 and 1.9 V *vs*. Li^+^/Li during the cathodic scan, and a double oxidation signal extending from 2.3 to 2.6 V *vs*. Li^+^/Li during the subsequent anodic scan. This response matches the multi‐step electrochemical process between lithium and sulfur, which leads to the formation of polysulfides of various chain‐length by the ongoing of discharge, and their conversion back upon charge.[^39^, ^40^] In addition, the CV signature excludes additional contribution to the electrochemical process of the Li‐(de)insertion reaction of MnO_2_, as indicated in literature.^[15]^ The decrease of the peak intensity in the CV of Figure 2a can be attributed to possible changes of the lithium ion diffusion coefficient,^[41]^ or partial loss of active material during cycling, which is particularly relevant in the conventional liquid ether‐based electrolyte that typically favors the dissolution and diffusion of the polysulfide intermediates. Nonetheless, the voltammograms present notable overlapping after the first cycles, thus indicating the stabilization of the interphase and remarkable reversibility of the Li−S conversion process after the initial stage. On the other hand, the kinetics of the electrochemical conversion may slow‐down both by the decrease of Li^+^ diffusion coefficient and by the limitation of the polysulfide anions mobility at the electrode/electrolyte interphase, due to electrolyte viscosity change promoted by Li‐PS dissolution. Therefore, the electrolyte optimization may actually speed up the reaction and improve the cell performances in terms of rate capability and capacity. Electrochemical impedance spectroscopy (EIS) is performed at the open circuit voltage condition upon cell assembly (OCV), and after 1, 5 and 10 CV runs, and the related Nyquist plots are displayed in Figure 2b. The plots are analyzed by non‐linear least squares (NLLS) fitting method to obtain the corresponding equivalent circuits formed by resistive (R) and constant phase elements (CPE, Q), identified by the R_e_(R_i_Q_i_)Q_w_ model in Table 1.[^42^, ^43^] In detail, R_e_ is the electrolyte resistance, identified by the high‐frequency intercept in the Nyquist plots, R_i_ and Q_i_ parallel elements (R_i_Q_i_) represent the single medium‐high frequency semicircles accounting for the electrode/electrolyte interphase, while Q_w_ indicates the semi‐infinite Warburg‐type Li^+^ ions diffusion which is observed as a tilted line at low frequency. Relevantly, the OCV plot in Figure 2b shows an almost vertical low‐frequency line attributed to the cell geometric capacity into quasi‐blocking electrode setup (Q_g_ in the corresponding equivalent circuit of Table 1). This response modifies in the subsequent Nyquist plots to a line tilted at about 45°, which is typical of a Warburg‐type semi‐infinite Li^+^ diffusion. Furthermore, Table 1 reveals a general increase of total interphase resistance of the S‐SM electrode (R_tot_, given by the sum of the R_i_ elements) from 78.6±3.6 Ω at the OCV to around 75.3±0.3 Ω after 1 CV run, and to 97.2±0.9 Ω after 10 cycles. The rise of the cell impedance can in part justify the slight peak‐current decrease discussed above in the corresponding CV of Figure 2a. The electrochemical performances of the S‐SM electrode are also investigated through galvanostatic tests in half‐cell at current rates increasing from C/10 (1 C=1675 mA g_S_^−1^) to C/8, C/5, C/3, C/2, 1 C and 2 C (Figure 2c and d), and at the constant rates of C/3 (Figure 2e) or 1 C (Figure 2f) for 250 cycles. The cycling trend depicted in Figure 2c for the test at increasing current rates shows a steady‐state discharge capacity taken at the 3^rd^ cycle for each C‐rate of 769, 756, 715, 670, 627, and 585 mAh g_S_^−1^ at C/10, C/8, C/5, C/3, C/2 and 1 C respectively. The delivered capacity drops below 200 mAh g_S_^−1^ at 2 C, thus suggesting this C‐rate as the limiting value for the cell application within the thin‐film configuration adopted in this work, in which a carbon coated Al collector with thickness of ~60 μm or lower is used. The cell recovers the relevant capacity value of 818 mAh g_S_^−1^ when the C‐rate is lowered back to C/10, thus indicating an excellent stability of the electrode by switching from high to low currents. The decrease of the cell capacity by increasing the current from C/10 to 1 C may be attributed to the rise of the discharge/charge polarization observed in the corresponding voltage profiles of Figure 2d, mainly due to kinetic limits and ohmic‐drops affecting the voltage plateaus evolving both at ~2.3 and ~2.1 V. On the other hand, a further increase of the current to 2 C turns into the deactivation of the electrochemical process related to the formation of short‐chain polysulfide at low voltages due to excessive polarization, and into a concomitant drop of the delivered capacity to a value reflecting a partial Li−S conversion to long‐chain polysulfide only (i. e., Li_2_S_8_). Despite the rate‐capability limit, the S‐SM electrode exhibits at C/3 (Figure 2e) a cycle life extended to 250 cycles with initial capacity of ~1090 mAh g_S_^−1^ retained for ~50 % at the end of the test, and a Coulombic efficiency (CE) approaching 98 % at the steady state. The fluctuations of the CE around 100 cycles may be attributed to changes and stabilizations of the passivation layers formed at the electrode/electrolyte interphases, likely driven by slight modifications of the reaction kinetics or by minor temperature fluctuations which have only marginal effects on the delivered capacity of the cell. The same cycle life and CE are exhibited at 1 C (Figure 2f), however with a different trend which involves a low initial capacity of ~330 mAh g_S_^−1^, increasing to ~500 mAh g_S_^−1^ over 20 cycles, and then fluctuating between these two values until the end of the test, with a retention of 72 % calculated as the ratio between the value after 250 cycles and the highest one achieved. The long‐term capacity reduction observed at C/3 could be attributed to a progressive loss of the soluble Li‐PS which can diffuse to the lithium metal, triggered by potential or concentration gradient, and precipitate on its surface.^[44]^ This decay can also be justified by the increase of the polarization with the ongoing of the cycles observed in the respective voltage profiles in Figure S3a (Supporting Information). Instead, the performance at 1 C may be rationalized by considering the relevant impact of the high C‐rate on cell overvoltage (Figure S3b), in view of the modest conductivity of the pristine sulfur in the electrode, which leads to a low initial capacity, rising upon cycling as the sulfur reacts with lithium and the conductivity improves. The subsequent capacity fluctuation can be ascribed to the combination of the two above discussed phenomena, i. e., a decrease due to the polysulfide precipitation by long‐term cycling and a rise as the electrode conductivity increases.[^14^, ^15^, ^16^] It is worth noting that the areal capacity normalized to the electrode geometric area (1.54 cm^2^ in our cells) represents a key parameter for evaluating electrochemical devices designated for energy storage.^[36]^ The previously discussed half‐cells retain a capacity >1.0 mAh cm^−2^ upon long term cycling as mainly due to the use of suitable cathode additives and current collector for compensating the negligible conductivity of sulfur, and simultaneously enabling a favorable rearrangement of the material upon cycles.[^16^, ^37^, ^45^] These results suggest the S‐SM electrode as suitable candidate for Li‐ and Li‐ion cell application. In particular, Li‐ion configuration is considered the most desirable version of the cell since it can mitigate the issues ascribed to the reactivity of the lithium metal.

**Table 1:** NLLS analysis carried out on the Nyquist plots displayed in Figure 2b. The analysis is performed on the impedance spectra acquired on the Li|S‐SM half‐cell at the OCV, and after 1, 5, and 10 CV runs using the Boukamp software, by exclusively accepting fits with χ^2^ values of the order of 10^−4^ or lower.[^42^, ^43^]

Cell condition	Circuit	R_1_ [Ω]	R_2_ [Ω]	R_i_ ( 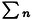 ) [Ω]	χ^2^
OCV	R_e_(R_1_Q_1_)(R_2_Q_2_)Q_g_	64.6±1.4	14.0±2.2	78.6±3.6	4×10^−5^
After 1 CV run	R_e_(R_1_Q_1_)Q_w_	75.3±0.3	/	75.3±0.3	1×10^−4^
After 5 CV runs	R_e_(R_1_Q_1_)Q_w_	90.6±0.6	/	90.6±0.6	2×10^−4^
After 10 CV runs	R_e_(R_1_Q_1_)Q_w_	97.2±0.9	/	97.2±0.9	3×10^−4^

**Figure 3:**
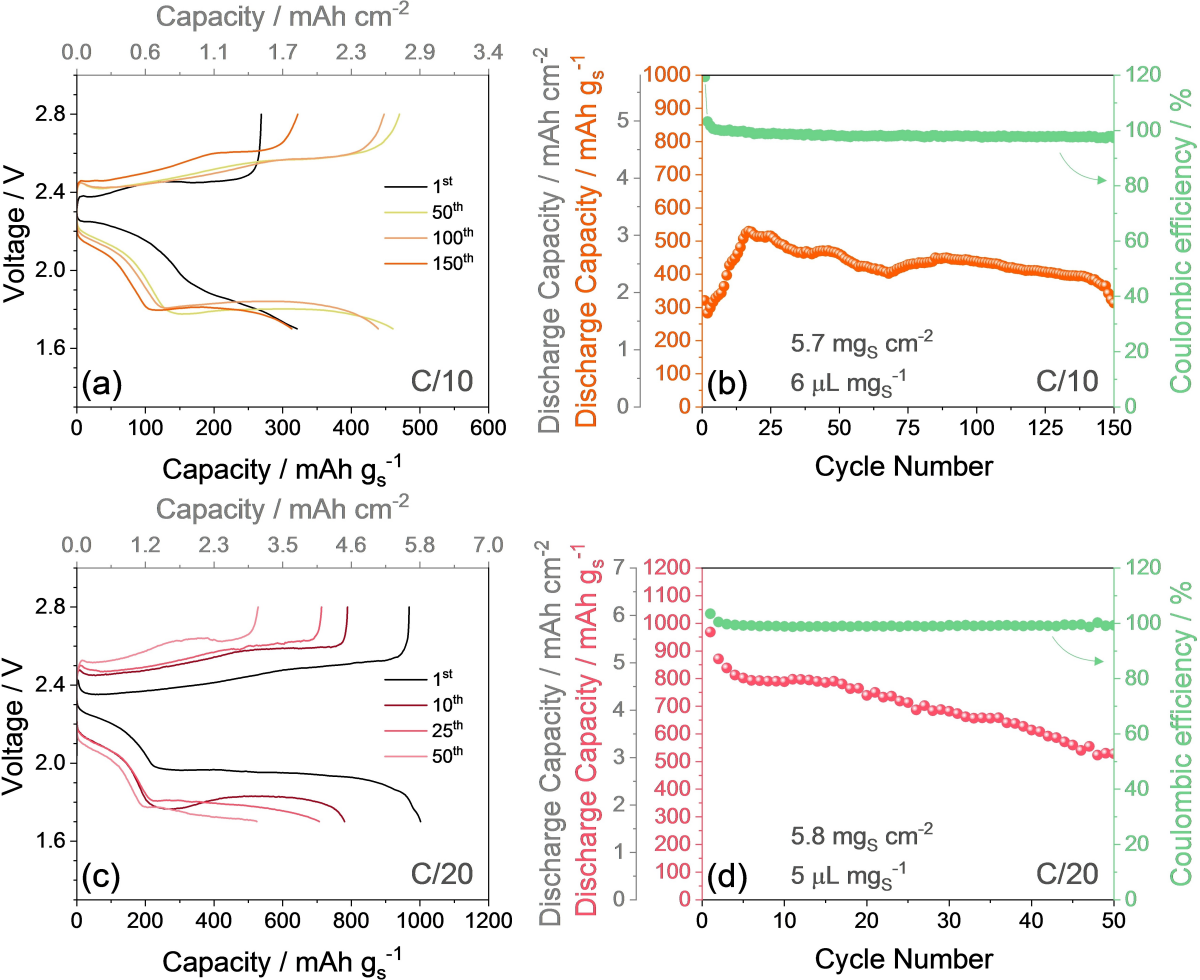
Galvanostatic cycling performances of the S‐SM electrode with high S‐loading in Li half‐cells with low E/S ratio, using the DOL:DME, 1 mol kg^−1^ LiTFSI, 1 mol kg^−1^ LiNO_3_ electrolyte. (**a**) Selected voltage profiles of a cell cycled at C/10 with sulfur loading of 5.7 mg cm^−2^, E/S ratio of 6 μL mg^−1^ (additional top *x*‐axis displays the areal capacity), and (**b**) corresponding cycling trend (right *y*‐axis shows CE, additional left *y*‐axis displays the areal capacity). (**c**) Selected voltage profiles of a cell cycled at C/20 with sulfur loading of 5.8 mg cm^−2^, E/S ratio of 5 μL mg^−1^ (top *x*‐axis display the areal capacity), and (**d**) corresponding cycling trend (right *y*‐axis shows CE, additional left *y*‐axis exhibits the areal capacity). Electrode geometric area: 1.54 cm^−2^. Voltage range: 1.7–2.8 V. Tests at 30 °C.

The actual role of Sn and MnO_2_ in the electrochemical performance of the S‐SM composite has been investigated by performing a galvanostatic cycling test on a Li−S half‐cell exploiting the bulk sulfur control electrode without any additive (see Experimental section). Figure S4 in the Supporting Information shows the cycling data recorded at C/3 rate in terms of selected voltage profiles (Figure S4a), and corresponding capacity and CE trends (Figure S4b). The first voltage profile in Figure S4a reveals a single discharge plateau centered at 2.2 V reversed into a corresponding charge process approaching 2.5 V, indicating the incomplete conversion reaction of S to Li‐PS likely due to the relevant insulant character of the electrode that leads to a delivered discharge capacity limited to 250 mAh g_S_^−1^. The subsequent profiles display the development of a highly polarized low voltage plateau during discharge below 1.8 V, and the evolution of merged charge processes between 2.4 and 2.5 V, as expected by the gradual lithiation of the sulfur electrode with concomitant increase of conductivity. However, the 100^th^ cycle shows the almost total disappearance of the low voltage discharge plateau and a final capacity limited to 230 mAh g_S_^−1^. In this regard, the capacity trend displayed in Figure S4b shows an activation of the delivered capacity in line with the gradual evolution of the low voltage discharge plateau which, on the other hand, allows a maximum capacity approaching 360 mAh g_S_^−1^ after 60 cycles that rapidly decreases to the final value of 230 mAh g_S_^−1^ (i. e., a capacity retention of 64 % after just 40 cycles). Despite the CE exceeds 99 % upon activation, the limited performance of the S control electrode in Li half‐cell evidences the advantages deriving by the addition of conducting Sn and the polysulfide‐retaining MnO_2_, as indeed demonstrated by the S‐SM composite which delivers higher capacity and longer cycling life also at the relevant rate of 1 C, exploiting in addition a slightly higher sulfur loading (compare with Figure 2). The areal capacity and energy density can be further improved by using S‐SM electrodes with increased sulfur‐loading and decreased E/S ratio as displayed in Figure 3. The figure shows the galvanostatic cycling tests performed in half‐cell using an electrode with sulfur loading of 5.7 mg cm^−2^ and E/S ratio of 6 μL mg_S_^−1^ at C/10 (Figure 3a and b), and into a more challenging E/S condition with sulfur loading of 5.8 mg cm^−2^ and E/S ratio of 5 μL mg_S_^−1^ at C/20 (Figure 3c and d). The related voltage profiles (Figure 3a and c) reveal at the first cycle (black curves) a discharge plateau between 2.4 and 1.9 V with associated capacity of 150–200 mAh g_S_^−1^. The cell cycled at C/10 (Figure 3a) shows a limited capacity during the subsequent plateau below 1.9 V, with an overall value of ~300 mAh g_S_^−1^ for the whole discharge. Instead, the cell cycled at C/20 evolves according to the typical voltage shape with a total capacity of ~1000 mAh g_S_^−1^ (Figure 3c). This difference can be mostly attributed to kinetic limits, which are more relevant at higher C‐rates due to excessive cell polarization. Hence, the cell cycled at C/10 progressively activates improving its voltage signature, where the capacity concomitantly rises to a maximum value of ~550 mAh g_S_^−1^, and then stabilizes with slight fluctuations until the end of the test (Figure 3b).^[7]^ The initial capacity increase during cycling of Li|S‐SM cells is attributed to the gradual lithiation of the active material with formation of Li‐PS that increases both ionic and electronic conductivity of the interphase, in concomitance with a cathode rearrangement that allows a better wetting and contact between the active material and the carbon‐based substrate of the current collector.^[14]^ This behavior is particularly evident in Figures 2f and 3b, due to the relatively high current rate of 1 C (Figure 2f) and the high sulfur loading (Figure 3b) that severely affect the kinetics of the Li−S conversion process. On the other hand, the cell cycled at C/20 undergoes partial deactivation of the electrochemical process, with increasing polarization in the related voltage profiles (Figure 3c) likely due to progressive precipitation of the dissolved polysulfide at the Li surface, which is promoted by the longer time requested for charge/discharge evolution at the lower current. At the end of the tests, the cells cycled at C/20 and C/10 deliver capacities of 528 and 313 mAh g_S_^−1^, retaining respectively 55 % and 59 % of the maximum achieved values, with a CE approaching 99 %. The CE is calculated in this work according to the equation $CE=\frac{CD}{CC}\times 100$ , i. e., the one typically used for Li‐ion battery, where *CD* is the discharge capacity and *CC* is the charge one. It is worth mentioning that the irreversible processes in Li‐ion battery typically occur during charge which has indeed higher capacity than discharge, thus leading to CE lower than 100 %. Instead, Li−S battery can occasionally reveal irreversible processes during discharge rather than charge, with a resulting CE over 100 %. Hence, the first cycle of the cell in Figure 3b shows partial reduction of the electrolyte during discharge, involving the formation of passivation layers on the electrodes surface, and incomplete oxidation of Li‐PS to sulfur during the charge which evolves with lower capacity and leads to a CE of ~120 %. Instead, Figure 3d indicates that the initial CE is limited to 103 % by lowering the current rate to C/20 and the E/S ratio to 5 μL mg^−1^. Furthermore, the difference observed in the capacity trends reported in Figure 3b and d can be likely attributed to the different cycling rates, that is, C/10 corresponding to ~1.0 mA cm^−2^ (Figure 3b) and C/20 corresponding to ~0.5 mA cm^−2^ (Figure 3d) for the adopted sulfur loading. Thus, the application of C/10 rate and the concomitant use of an areal sulfur loading as high as 6 mg_S_ cm^−2^ leads to kinetic limits extending over the initial 20 cycles requested to reach the maximum capacity of 550 mAh g_S_^−1^ (Figure 3b), while the use of the relatively lower current of C/20 allows an efficient exploitation of the Li−S conversion process already at the first cycle with maximum discharge capacity of ~1000 mAh g_S_^−1^ (Figure 3d). It is worth mentioning that the voltage profile during the 1^st^ cycle of the cell with high S‐loading and low E/S ratio at C/10 (i. e., 5.7 mg_S_ cm^−2^ and 6 μL mg_S_^−1^ in Figure 3a) differs from the one observed for the cell with lower S‐loading and higher E/S ratio at 1 C (i. e., 2.9 mg cm^−2^ and 10 μL mg_S_^−1^ in Figure S3b). The reason for this discrepancy may be found in the different nature of the kinetic limits hampering the two measurements, i. e., elevated S content and low electrolyte ratio in the former and high C‐rate in the latter. Instead, the related cycling trends appear comparable (compare Figure 3b and Figure 2f) due to the analogue effects of the kinetic limits, which hinder the Li−S conversion process and depress the delivered capacity.^[16]^ The top *x*‐axes in Figure 3a and c and the additional left *y*‐axes in Figure 3b and d suggest for the half‐cells using the S‐SM electrode a maximum areal capacity of 3.0 mAh cm^−2^ and a stabilized value of ~2.5 mAh cm^−2^ at C/10, while values ranging from 6.0 to 3.0 mAh cm^−2^ are observed at C/20.^[23]^ These features, in addition to the limited overall thickness of the S‐SM electrode (~100 μm), may certainly favor the achievement of practical cells. However, further researches on suitable electrolytes and anodes are requested to possibly achieve a large‐scale diffusion of energy storage systems using sulfur as the active material.[^10^, ^21^]

**Figure 4:**
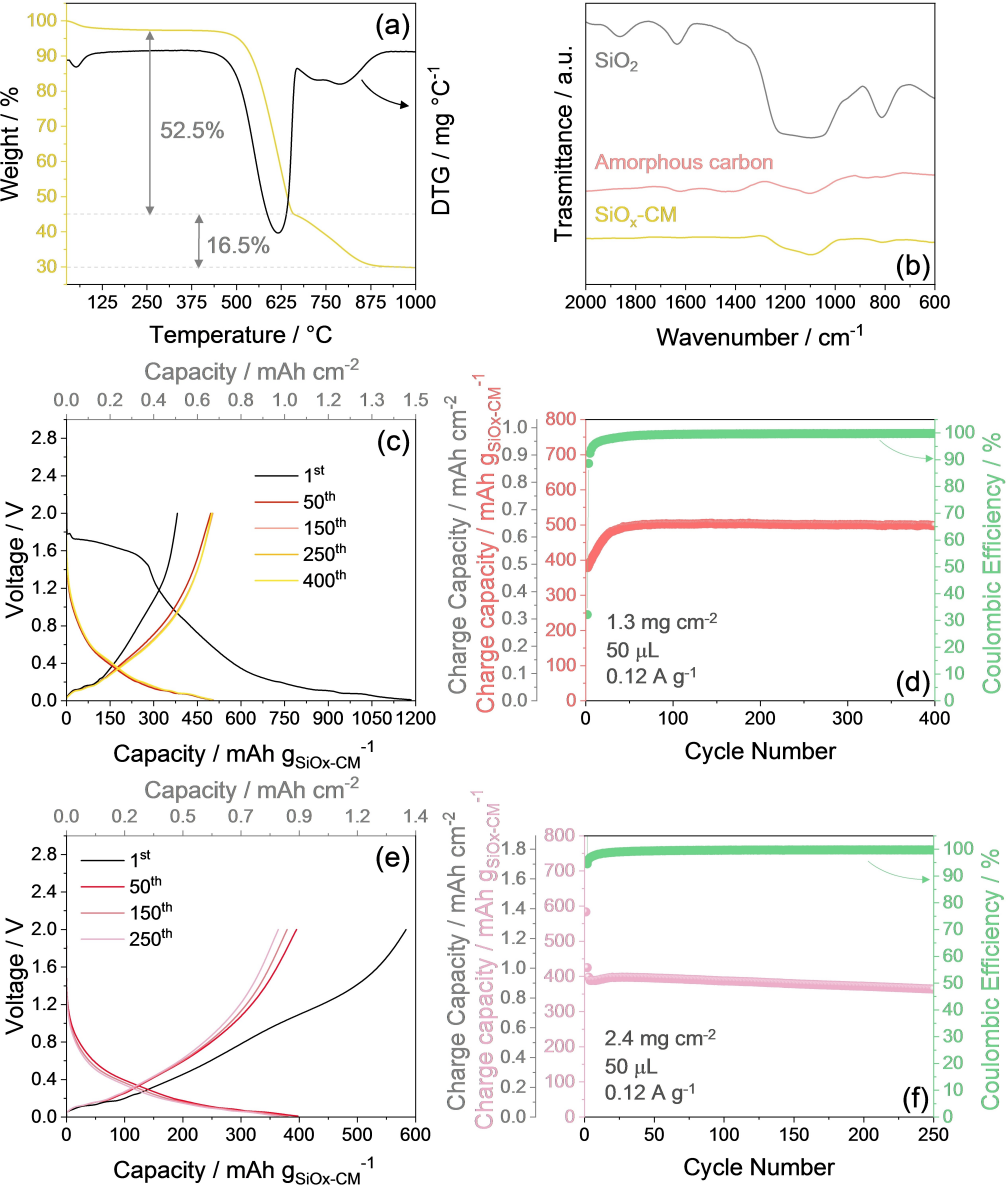
(**a** and **b**) Brief physical‐chemical characterization of the SiO_x_‐CM powder and (**c**–**f**) electrochemical features of the electrode in Li half‐cell using the DOL:DME, 1 mol kg^−1^ LiTFSI, 1 mol kg^−1^ LiNO_3_ electrolyte, applying a current of 120 mA g^−1^. (**a**) TGA (yellow) performed under dry air flow with heating rate of 5 °C min^−1^ from 25 to 1000 °C and corresponding DTG (black). (**b**) FTIR spectra of SiO_2_ (grey), amorphous carbon (red), and SiO_x_‐CM (yellow). (**c**) Selected voltage profiles of the galvanostatic cycling test of the SiO_x_‐CM electrode (top *x*‐axis shows areal capacity), and (**d**) corresponding cycling trend (right *y*‐axis shows CE, additional left *y*‐axis displays areal capacity). (**e**) Selected voltage profiles of the cycling tests of the chemically lithiated Li_y_SiO_x_‐CM electrode (top *x*‐axis shows areal capacity), and (**f**) corresponding cycling trend (right *y*‐axis shows CE, additional left *y*‐axis exhibits areal capacity). Tests performed at 30 °C. Voltage range: 0.01–2.0 V. Electrodes geometric area: 1.54 cm^2^.

Recently, Li‐ion sulfur battery arose a great interest since it can suppress issues ascribed to the metallic lithium, such as dendrite formation, short‐circuit hazards, and shuttle process.[^29^, ^46^, ^47^] We propose subsequently a SiO_x_‐ composite material (CM) anode developed according to our previous work,^[35]^ and *ad hoc* lithiated herein to achieve a Li_y_SiO_x_‐CM version suitable to act as the lithium source into a Li‐ion configuration using the S‐SM cathode. Since SiO_x_‐CM is newly synthesized in this work with a lower silica content than the previous composite, the principal physical‐chemical and electrochemical characteristics of the material are double‐checked in Figure 4 prior to use in full‐cell. The TGA curve under air of the SiO_x_‐CM reported in Figure 4a displays amounts of amorphous carbon and FLG of 52.5 % and 16.5 % respectively, while the silica content can be calculated as the residual weight of 31 %.^[35]^ The oxidative loss of amorphous carbon is indicated in the corresponding DTG curve by the peak centered at 615 °C, while the crystalline FLG loss is detected by the wide peak centered at 780 °C.^[35]^ Figure 4b reports the Fourier‐transform infrared (FTIR) spectrum of SiO_x_‐CM (black curve), in comparison with the spectra of amorphous carbon (red curve) and SiO_2_ (yellow curve) blanks, while FLG is neglected due to relatively limited IR activity.^[48]^ The figure principally reveals for the anode composite the presence of a broad band between 1300 cm^−1^ and 1000 cm^−1^ accounting for the Si−O−Si stretching vibration, also observed in the SiO_2_ blank spectrum,^[49]^ whereas the contribution of carbon appears very modest. The FTIR spectrum of SiO_x_‐CM thus confirms the actual presence of SiO_2_ and its partial retention during synthesis of the material, despite the use of a chemically reducing environment (Ar/H_2_) during the process (see Experimental section). The SiO_x_‐CM electrode is cycled in lithium half‐cell at 120 mA g^‐1^, and the related voltage profiles and cycling trends are depicted in Figure 4c and d, respectively. The signature of Figure 4c indicates an irreversible multi‐step discharge during the first cycle (black curve) evolving between 1.7 and 0.4 V, due to partial reduction of the electrolyte with SEI film formation,[^22^, ^50^] and partial conversion of SiO_x_ to Li_2_O and Si.^[35]^ This side process is followed by a voltage slope below 0.4 V, accounting for the insertion/intercalation of lithium into the carbons as well as partial Li−Si alloying.^[51]^ The subsequent cycles reveal a reversible electrochemical feature between 0.1 and 0.4 V with notable overlapping and low polarization, where the various merged plateaus both during charge and discharge are ascribed to the Li−Si (de)alloying, Li‐(de)insertion with amorphous carbon, and Li‐(de)intercalation within FLG.^[35]^ The related cycling trend in Figure 4d shows an initial capacity of 380 mAh g^−1^, which remarkably improves after 15 cycles to a steady‐state value of 500 mAh g^−1^ held for over 400 cycles with impressive stability. The increase of the delivered capacity during the initial cycling stages of the Li|SiO_x_‐CM half‐cell observed in Figure 4d can be ascribed to the progressive rearrangement of the electrode structure including lithiation and alloying of the Si/SiO_2_ within the electrode, as well as to the formation and subsequent stabilization of the SEI at the electrode/electrolyte interphase, as indeed observed by CV and EIS in the previous study.^[35]^ On the other hand, the use of the SiO_x_‐CM anode in a Li‐ion cell in combination with the S‐SM cathode mandatorily requests a pre‐lithiation process, since the latter electrode is Li‐free in its pristine state. The lithiated Li_y_SiO_x_‐CM phase may be achieved electrochemically by cycling the SiO_x_‐CM electrode in half‐cell for few runs (e. g., the initial stages of Figure 4c and d), holding the voltage at the lowest value, and then retrieving the electrode for the subsequent use in the full‐cell. As an alternative, the lithiation of the anode can be also obtained chemically through direct reduction upon mechanical contact under pressure of the electrode with a lithium foil soaked in the electrolyte, and its removal after a time sufficient to reach the lithium content necessary for allowing adequate conversion process at the sulfur cathode.^[46]^ This would satisfy the above mentioned need for a Li reservoir in the full‐cell, since both the SiO_x_‐CM and S‐SM pristine composites do not contain any lithium necessary to trigger the Li−S conversion process, which is instead easily developed in S‐based cells using a Li‐metal anode. Figure 4e reports the voltage profiles at 120 mA g^−1^ of a Li_y_SiO_x_‐CM electrode previously chemically lithiated, while the corresponding cycling trend is shown in Figure 4f. The first charge profile in Figure 4e (de‐lithiation, black curve) evidences that the chemical treatment leads to a capacity exceeding the one of the subsequent cycles, mostly due to an excessive lithium‐uptake in the electrode promoted by treatment. This excess rapidly vanishes, and the subsequent cycles evolve with a steady‐state volage centered at 0.4 V and an excellent stability over 250 cycles, with CE exceeding 99 % after the initial cycles (Figure 4f). However, the chemically lithiated Li_y_SiO_x_‐CM reveals a lower steady‐state capacity than the pristine electrode (compare Figure 4f and d), likely due to minor material loss by detachment during the lithiation process which foresees some mechanical stress (see Experimental section for further details). These results suggest the suitability of both electrochemical and chemical lithiation herein exploited for achieving efficient anodes for full‐cell application with sulfur cathodes, and indicate that further efforts on the chemical lithiation process can lead to even better results and more relevant scalability of the full‐cell.[^52^, ^53^]

**Figure 5:**
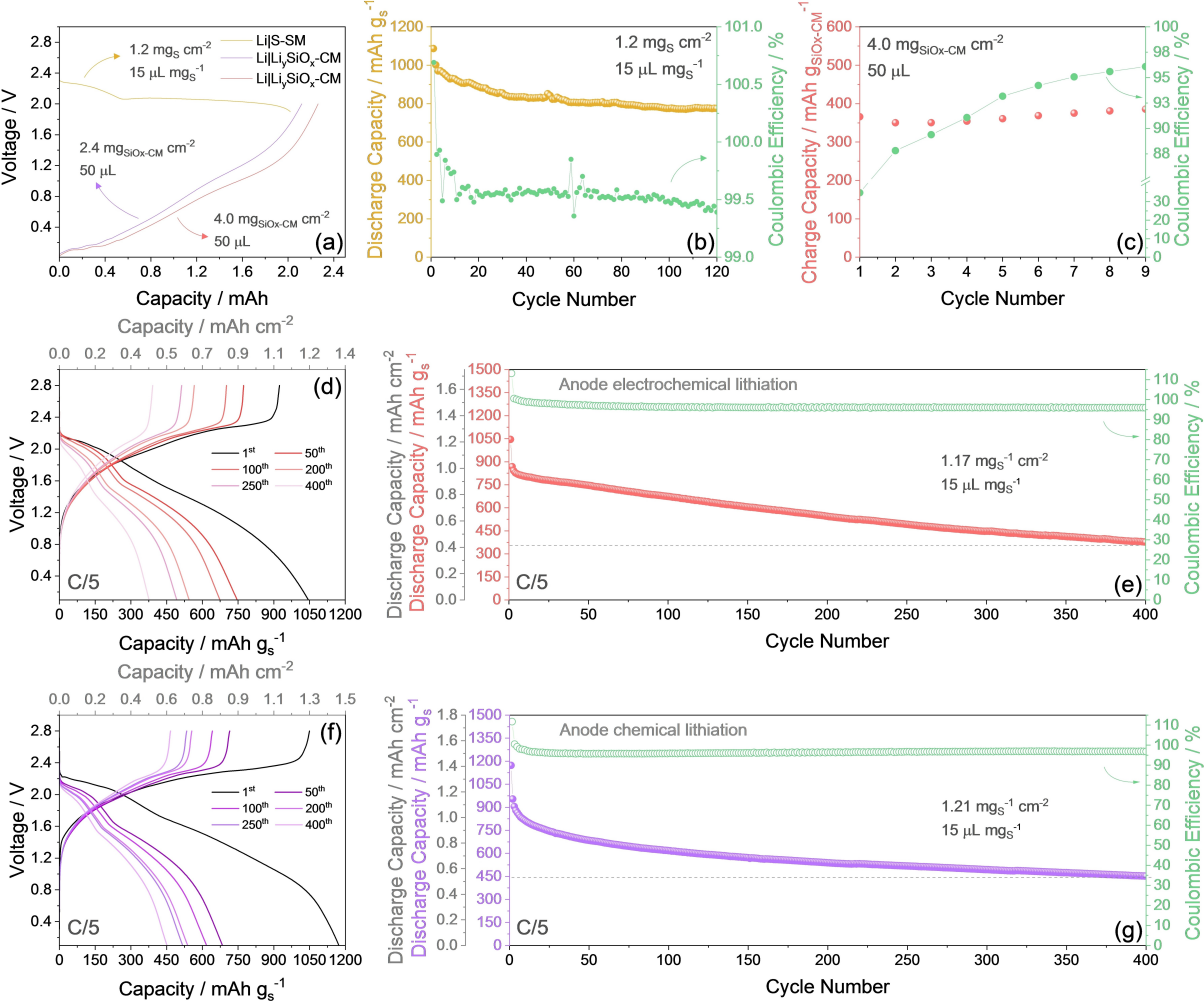
(**a**–**c**) Examples of chemical and electrochemical lithiation of a Li_y_SiO_x_‐CM anode for determining the N/P ratio of a full‐cell with S‐SM cathode. (**a**) Charge (de‐lithiation) profiles in lithium half‐cells until 2.0 V of Li_y_SiO_x_‐CM chemically lithiated for 14 h (purple) and electrochemically lithiated (red), respectively at 120 mA g^−1^ and 20 mA g^−1^, and discharge profile (Li−S conversion) of S‐SM (yellow) in lithium half‐cell at C/5 until 1.9 V. (**b**) Cycling trend of S‐SM in lithium half‐cell at C/5 between 1.9 and 2.8 V (right *y*‐axis displays CE). (**c**) Cycling trend of the electrochemical lithiation of Li_y_SiO_x_‐CM in lithium half‐cell at 20 mA g^−1^ between 0.01 and 2.0 V (right *y*‐axis displays CE). (**d**–**g**) Galvanostatic cycling performance of the Li_y_SiO_x_‐CM|S‐SM full cell at C/5 (1 C=1675 mA g_S_^−1^) in the voltage range of 0.1–2.8 V. (**d**) Test performed using the electrochemically lithiated Li_y_SiO_x_‐CM in terms of selected voltage profiles (top *x*‐axis shows areal capacity), and (**e**) corresponding cycling trend (right *y*‐axis shows CE, additional left *y*‐axis displays areal capacity). (**f**) Test performed using the chemically lithiated Li_y_SiO_x_‐CM in terms of selected voltage profiles (top *x*‐axis shows areal capacity), and (**g**) corresponding cycling trend (right *y*‐axis shows CE, additional left *y*‐axis displays areal capacity). Dashed lines in panels e and g indicate areal and specific capacity at the end of the tests. All tests performed at 30 °C. Electrodes geometric area: 1.54 cm^2^. Electrolyte: DOL:DME, 1 mol kg^−1^ LiTFSI, 1 mol kg^−1^ LiNO_3_. See Experimental section for chemical and electrochemical lithiation of the pristine SiO_x_‐CM electrode.

Figure 5 exemplifies the features of the chemically and electrochemically lithiated Li_y_SiO_x_‐CM anodes in lithium half‐cells and shows their subsequent combination in Li‐ion full‐cells using the S‐SM cathode. The charge (de‐lithiation) profiles in half‐cell of Li_y_SiO_x_‐CM chemically lithiated (purple) and electrochemically lithiated (red) reported in Figure 5a reveal the expected shape evolving mainly below 1.2 V, and a delivered capacity exceeding 2.0 mAh which is suitable for allowing the full discharge (Li−S conversion) of a S‐SM cathode with a sulfur loading of ~1.2 mg_S_ cm^−2^, such as in the example reported in yellow in the same panel for comparison. Furthermore, the cycling trends in half‐cell of the S‐SM (Figure 5b) and of the electrochemically lithiated Li_y_SiO_x_‐CM (Figure 5c) allow the rough estimation of the N/P ratio in a full‐cell that combines the two electrodes. Indeed, Li_y_SiO_x_‐CM|S‐SM full‐cells using chemically and electrochemically lithiated anodes are assembled with an initial N/P ratio of ~1.05 and ~1.12, respectively, calculated by considering the half‐cell capacity of S‐SM discharge (i. e., 2.02 mAh) and Li_y_SiO_x_‐CM charge (i. e., 2.12 and 2.26 mAh) in Figure 5a. On the other hand, we can predict that these N/P ratios may rise‐up by comparing with the examples of the half‐cells exploiting S‐SM (Figure 5b) and Li_y_SiO_x_‐CM (Figure 5c), as the anode undergoes progressive interphase improvement and its capacity increases.[^46^, ^47^] The voltage profiles of the cycling test performed at C/5 (1 C=1675 mA g_S_^−1^) on a full‐cell combining S‐SM and electrochemically lithiated Li_y_SiO_x_‐CM is reported in Figure 5d, while the corresponding capacity trend and respective CE are displayed in Figure 5e. The voltage profile of the first cycle (black curve in Figure 5d) appears as the combination between the double‐plateau associated with the multi‐step conversion process of sulfur with lithium, and the sloped curve ascribed to the Li^+^ (de)alloying/(de)insertion reaction of the SiO_x_‐CM. Indeed, the cell exhibits two broad discharge plateaus centered around 2 V and 1.7 V, reversed into a merged charge profile evolving between 1.2 V and 2.4 V, with an initial reversible capacity of 1045 mAh g_S_^−1^. The corresponding cycling trend (Figure 5e) shows an immediate drop of the capacity from 1050 to 980 mAh g_S_^−1^, and subsequently a progressive decrease to 380 mAh g_S_^−1^ upon 400 cycles, with CE exceeding 95 % after the first run. The observed capacity decay is likely ascribed both to the incomplete de‐alloying of the Li−Si phases during discharge at the anode upon repeated cycles and to the loss of active material, i. e. sulfur, due to deposition of Li‐PS at the anode side. A very similar voltage shape is observed for the full‐cell using the chemically lithiated Li_y_SiO_x_‐CM (Figure 5f), however with a higher discharge capacity during the first cycle, i. e., 1170 mAh g_S_^−1^. In addition, the corresponding cycling trend (Figure 5g) shows a more relevant loss during the initial cycles, while a slightly higher efficiency and stability upon prolonged test, with a final capacity of 450 mAh g_S_^−1^ after 400 cycles. Despite the final capacities delivered by the full Li‐ion sulfur cells of 380 and 450 mAh g_S_^−1^, respectively, correspond to a retention limited to about 40 %, it is worth considering that they still represent considerable values if compared to those of commercial Li‐ion cells using the common insertion/intercalation cathodes and the graphite anode. Therefore, the Li‐ion‐sulfur cells we propose herein are a first‐look to a proof‐of‐concept system that might represent, upon extended optimization, a competitive energy storage strategy based on the challenging Li−S conversion mechanism while relying on sustainable materials and a non‐reactive anode. These results are certainly encouraging, in particular by considering the long cycling evolution, the absence of relevant signs of dendrites, and the possible scalability of the cell using the chemically lithiated Li_y_SiO_x_‐CM. Furthermore, the practical energy density of the cell is estimated to initially exceed 500 Wh kg^−1^. However, the capacity decay observed by long‐term cycling in Figure 5, and the corresponding limitation of the practical energy density, suggest the necessity of additional improvement for scaling‐up the system, such as a better tuning of the N/P ratio and the increase of the active material loading to achieve more appealing areal capacity values.

**Figure 6:**
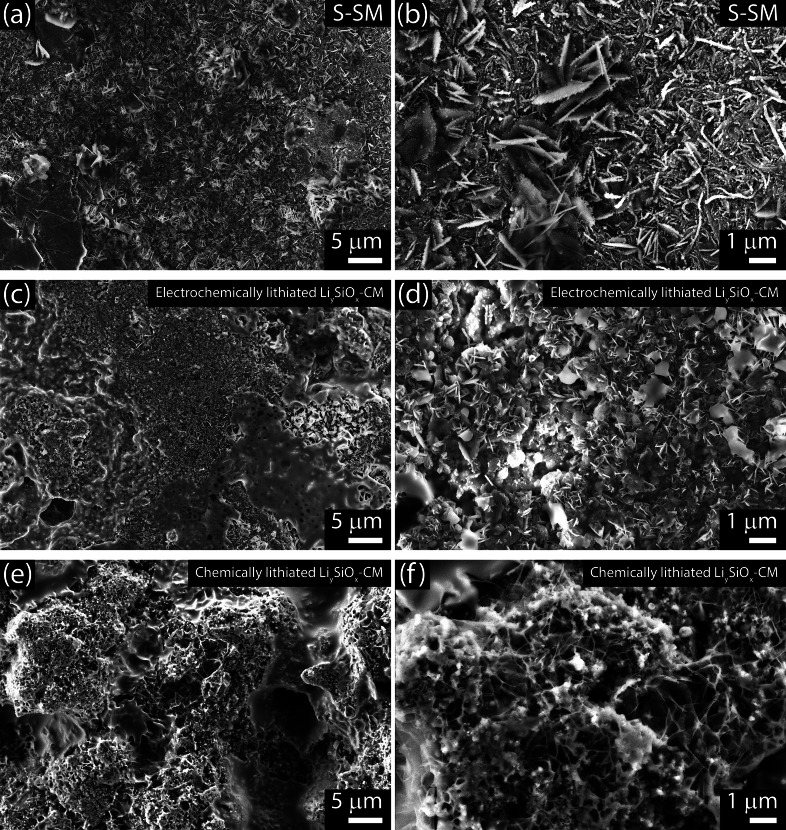
*Ex‐situ* SEM performed on the electrodes extracted from full Li_y_SiO_x_‐CM|S‐SM cells after 400 galvanostatic cycles (see corresponding cells in Figure 5d–g). (**a** and **b**) S‐SM cathode (from test in Figure 5d and e) and (**c**–**f**) Li_y_SiO_x_‐CM anode, either upon (**c** and **d**) electrochemical lithiation or (**e** and **f**) chemical lithiation.

Figure 6 shows the *ex‐situ* SEM images of the S‐SM and Li_y_SiO_x_‐CM electrodes after 400 cycles in the full‐cells illustrated in Figure 5d–g, performed to evaluate the morphological changes in response to prolonged galvanostatic cycling. The SEM micrographs related to the S‐SM cathode in Figure 6a and b show a still compact electrode morphology, where the micrometric FLG flakes are homogeneously dispersed in the MWCNTs network of the current collector substrate,^16^ while MnO_2_ and Sn particles^[14]^ can be hardly observed in line with their notably low contents adopted herein (i. e., 5 wt % each in the sulfur composite).^[15]^ In this regard, the bright spots observed in the surface may indicate the deposition of poorly conducting products such as Li_2_S upon cycling.^[14]^ Interestingly, the agglomerated sulfur already observed in the pristine composite (Figure 1f) almost vanishes from surface of the cycled electrode, which is in line with a massive dissolution and operation as a catholyte of the active material upon prolonged cycling, as demonstrated in previous work.^[54]^ The *ex‐situ* SEM images of Li_y_SiO_x_‐CM anodes (Figure 6c–f) show at the lower magnifications a more compact and flat surface for the electrochemically lithiated electrode (Figure 6c) than the chemically lithiated one (Figure 6e), possibly due to the physical stress promoted by pressing the latter during the pre‐treatment (see Experimental section). On the other hand, a higher magnification of the image reveals the presence of submicrometric precipitates in the electrochemically lithiated anode (Figure 6d), while the chemically lithiated one presents an amorphous deposit (Figure 6f). This difference may suggest a characteristic behavior of the deposition of the Li‐PS on the anode surface depending on the type of lithiation pathway adopted, which would also justify the different performance achieved by the cells in Figure 5. It is worth mentioning that the influence of the lithiation mechanism of SiO_x_‐CM on the performance of the Li‐ion sulfur can be also investigated with *ad hoc* studies focusing on the actual chemistry of the anode/electrolyte interphase.

The combination of the electrochemically lithiated Li_y_SiO_x_‐CM anode with the S‐SM cathode in full‐cell is further tested by exploiting more challenging conditions, namely, active material loadings of 6.30 mg cm^−2^ for the pristine SiO_x_‐CM and 2.90 mg cm^−2^ for S‐SM, as well as an E/S ratio limited to 8 μL mg^−1^, as reported in Figure S5 in Supporting Information. The selected voltage profiles and corresponding capacity trend in Figure S5a and b, respectively, recorded at decreasing rates of C/5, C/10 and C/20, display an initial capacity approaching 500 mAh g_S_^−1^ and final value of 340 mAh g_S_^−1^ after 280 cycles. The data indicate that the gradual lowering of the current rate upon cycling leads to an increase of capacity retention. Interestingly, the capacity decay experienced by the cell between 1^st^ and 2^nd^ cycle from 490 to 420 mAh g_S_^−1^ is far less relevant than those observed for the previous full‐cells (compare with Figure 5), which is likely due to the decreased E/S ratio that hinders excessive dissolution of the Li‐PS, despite it limits, at the same time, the delivered capacity. In addition, the relevant capacity retention is achieved even with a decrease of the CE, which shows values around 98 % that drop to 93 % at C/10 and, subsequently, to 84 % at C/20. This behavior is ascribed to the low cycling rates which trigger both partial electrolyte degradation and excessive dissolution and consequent diffusion of the Li‐PS from the relatively high loaded cathode resulting in the promotion of partial loss of active material. Nonetheless, the full Li_y_SiO_x_‐CM|S‐SM cell exploiting challenging conditions displays promising performance also promoted by proper electrochemical lithiation of the highly loaded anode, which was obtained through a limited number of cycles with respect to the tests displayed in Figure 5, i. e., 5 full charge/discharge runs instead of 9, as represented in Figure S5c in terms of voltage profiles (top panel) and areal capacity trend (bottom panel). Indeed, the comparison in Figure S5d of the full‐cells presented herein reveals a capacity retention for the high‐loaded full‐cell as high as 70 % after about 280 cycles, while the ones previously investigated exhibit values between 43 and 45 % at the same cycle number. These latest results fulfill the purpose of this work by demonstrating the effectiveness of our simple approach in achieving a promising full Li‐ion sulfur cell exploiting a sulfur loading approaching 3 mg cm^−2^, despite they also evidence the need of *ad hoc* improvements across the whole system, from the composition of electrodes and electrolyte for hindering the issues of Li‐PS to the cycling conditions.

## Conclusions

Li‐ion sulfur cells with a remarkable cycle life have been achieved by combining a composite sulfur cathode added with Sn and MnO_2_ (S‐SM) with a pre‐lithiated silicon oxide/carbon anode (Li_y_SiO_x_‐C). TGA and SEM‐EDS of the S‐SM cathode material evidenced that Sn and MnO_2_, with an overall weight ratio of 10 % in the composite, have been effectively included into micrometric sulfur aggregates with active material loading as high as 90 %. TEM showed the nanometric size of Sn used to promote the electrode conductivity, and the submicron shape of MnO_2_ which acted as a trap for the dissolved polysulfides. CV and EIS tests performed in lithium half‐cell suggested for the S‐SM cathode moderately fast Li−S conversion, and adequate conductivity of the electrode/electrolyte interphase. Specifically, the CV profiles identified two reversible discharge peaks at 2.2 and 1.9 V *vs*. Li^+^/Li, and a merged charge process extending from 2.3 to 2.6 V *vs*. Li^+^/Li, with interphase resistance measured by EIS of 78.6 Ω at the OCV increasing up to 97.2 Ω upon 10 voltammetry cycles. The S‐SM electrode exhibited in half‐cell a reversible electrochemical process with maximum capacity of ~1090 mAh g_S_^−1^, CE approaching 99 %, and a rate capability extending up to 1 C. Furthermore, the material showed a moderate capacity retention over 250 cycles, with a long‐term decay mostly due to dissolved polysulfides precipitation on the reactive lithium surface. Therefore, we have proposed as alternative an electrochemically or chemically pre‐lithiated Li_y_SiO_x_‐CM anode, which can achieve in half‐cell a maximum capacity of 500 mAh g^−1^ for over 400 cycles without significant decay. The full Li_y_SiO_x_‐CM|S‐SM cells revealed at C/5 a voltage signature centered at about 1.8 V, reflecting the combination of the multi‐step sulfur conversion and the (de)alloying/(de)insertion process of the anode, with initial capacity exceeding 1000 mAh g_S_^−1^. The Li‐ion batteries demonstrated a significant performance extended over 400 cycles, a CE higher than 95 % after the initial stages, however with capacity decay leading to a final value of 380 mAh g_S_^−1^ for the cell using the electrochemically lithiated anode and of 450 mAh g_S_^−1^ for the cell using the chemically lithiated one. These responses have been achieved by leveraging suitable anode and cathode, with a N/P ratio from 1.02 to 1.12 set to concomitantly allow long cycle life and remarkable efficiency. Furthermore, the cycling trends suggested that the stability of the cell can be improved by adequately adjusting the N/P ratio, and by setting up the active material loading. Thus, the use of relatively high active material loadings (i.e., 6.30 and 2.90 mg cm^−2^ for anode and cathode, respectively) and the limitation of the E/S ratio to 8 μL mg^−1^, alongside with optimized cycling conditions, improved the capacity retention, despite *ex‐situ* SEM of the electrodes upon cycling in full‐cells revealed dissolution of sulfur from the cathode and partial deposition of Li‐PS at the anode side. On the other hand, the use of thin‐layer cathode and anode, in addition to the chemical lithiation process, may represent a step forward for facilitating the practical scaling‐up of high‐energy Li‐ion batteries exploiting the sulfur chemistry.

## Experimental

### Synthesis of the S‐SM Composite

Elemental sulfur (≥99.5 %, Riedel‐de Haën), Sn (nanopowder, <150 nm particle size, ≥99 % trace metals basis, Sigma‐Aldrich), and MnO_2_ (≥99 %, ReagentPlus) were mixed in the 90 : 5 : 5 *w/w* ratio and heated at 125 °C under magnetic stirring in a silicon oil bath until complete melting of sulfur and uniform mixing with Sn and MnO_2_. The value of 125 °C was chosen to allow sulfur melting, which begins slightly over 110 °C in standard condition, and avoid at the same time its possible evaporation which may occur at higher temperatures and decrease the sulfur content in the composite.^[37]^ The mixture was subsequently quenched at room temperature until the complete solidification, and ground in an agate mortar to obtain a fine grey powder. The S‐SM amount produced by our simple physical mixing‐based preparation pathway exceeded 5 g at laboratory scale.

### Synthesis of SiO_x_‐CM

SiO_x_‐CM was synthetized as reported in our previous work.^[35]^ In brief, the synthesis involved a hydrothermal step and a subsequent annealing in Ar/H_2_ of a mixture of sucrose, FLG, and SiO_2_. The sucrose was used as the source of amorphous carbon in the final composite in combination with FLG to promote the contribute of the SiO_x_ to the electrochemical process by partial Li‐(de)alloying, as well as to act as active materials for the Li‐(de)insertion and Li‐(de)intercalation.^[35]^

### Materials Characterization

The S‐SM structure was investigated by XRD through a Bruker D8 Advance instrument equipped with a Cu K*α* radiation source (8.05 keV) scanning the 10°−50° 2*θ* range using a step size of 0.02° and a rate of 10 s step^−1^. TGA was performed in a Mettler‐Toledo TGA 2 instrument (Mettler‐Toledo, Columbus, OH, USA) under dry N_2_ (for S‐SM) or dry air (for SiO_x_‐CM) flow of 50 ml min^−1^, heating the samples from 25 to 400 or 1000 °C, respectively, with rate of 5 °C min^−1^. The morphology of S‐SM powder was detected through SEM using a Zeiss EVO 40 relying on a LaB_6_ thermionic gun in backscattered electrons mode, and by TEM through a Zeiss EM910 equipped with a tungsten thermionic electron gun operating at 100 kV. The sample for TEM analyses consisted of a suspension of S‐SM powder in ethanol which was drop‐cast onto a formvar/carbon supported copper grid (150 mesh). EDS elemental maps were collected on the SEM backscattered electrons images via a X‐ACT system associated with the SEM equipment. FTIR spectra were recorded via a Bruker Vertex V70 instrument set up in transmittance mode.

### Electrodes Preparation

The electrodes tapes were prepared by casting process with a doctor blade tool (MTI Corp.) set at ~300 μm of slurries formed by 80 wt % active material powder, namely S‐SM, S or SiO_x_‐CM, 10 wt % polyvinylidene fluoride (PVDF 6020, Solef) polymer binder, and 10 wt % few layer graphene (FLG, produced via WJM method by BeDimensional S.p.A.) in the cathodic slurry,^[55]^ or 10 wt % carbon black in the anodic slurry (super P carbon, SPC, Timcal) as electron conductor dispersed in N‐methyl‐2‐pyrrolidone (NMP, Sigma‐Aldrich). The slurries were cast on MWCNTs‐coated aluminum foil (thickness of 60 μm, prepared according to our previous work)^16^ for S‐SM and S or copper foil (thickness of 20 μm, MTI Corp.) for SiO_x_‐CM.^35^ The tapes were heated for 2 hours at 50 °C on a hot plate under a fume hood (air atmosphere) to remove the NMP solvent. The obtained foils were calendared at the 70 % with respect to the initial thickness using an MSK‐2150 Rolling Machine (MTI Corp.) to achieve a final thickness of ~100 μm, and cut into discs with diameter of 14 mm (1.54 cm^2^ geometric area) with a Nogami handheld punch. The SiO_x_‐CM electrodes were dried under vacuum for 3 h at 110 °C, while the S‐SM and S control electrodes were dried under vacuum overnight at 30 °C inside a Büchi oven, and subsequently stored inside an Ar‐filled glovebox (MBraun, O_2_ and H_2_O contents lower than 1 ppm). The electrodes had an active material loading ranging from 1.2 to 5.8 mg cm^−2^ for S‐SM and S (control) and between 1.3 and 6.3 mg cm^−2^ for SiO_x_‐CM (note that all active material loadings are repeated in the figure panels where the electrodes are subjected to cycling tests).

### Cell Assembly and Electrolyte Preparation

CR2032 coin‐type cells (MTI Corp.) were assembled inside an Ar‐filled glovebox by stacking a 14 mm‐diameter lithium metal disc (0.25 mm thickness, MTI Corp.) as counter electrode, an 18 mm‐diameter monolayer microporous membrane (Celgard 2500) as separator soaked with electrolyte (see composition below), and a S‐SM, S (control) or SiO_x_‐CM disc as working electrode. The electrolyte consisted of a solution formed by DOL (anhydrous, contains ca. 75 ppm BHT as inhibitor, 99.8 %, Sigma‐Aldrich) and DME (anhydrous, 99.5 %, inhibitor‐free, Sigma‐Aldrich) mixed in the 1 : 1 weight ratio, and dissolving LiTFSI (LiN(SO_2_)_2_(CF_3_)_2_, 99.95 % trace metals basis, Sigma‐Aldrich) as conducting salt and lithium nitrate (LiNO_3_, 99.99 % trace metals basis, Sigma‐Aldrich) as passivating agent in the solvents mixture with a 1 mol kg_solvent_^−1^ concentration for each salt. Before using, DOL and DME solvents were preserved under molecular sieves (rods, 3 Å, size 1/16 inch, Honeywell Fluka) until a H_2_O content lower than 10 ppm was achieved as determined by a Karl Fischer 899 Coulometer (Metrohm), while LiTFSI and LiNO_3_ were dried for 2 days under vacuum at 110 °C. A catholyte containing lithium polysulfide (Li_2_S_8_) for UV‐vis analyses was prepared by adding 0.5 wt % of Li_2_S_8_ to the above described electrolyte solution. The Li_2_S_8_ addition procedure has been also described in a previous work.^[56]^ UV‐vis analyses were carried out on the above catholyte reference, and on catholyte solutions aged for 1 hour in contact with 20 mg of Sn or 20 mg of Sn:MnO_2_ 1 : 1 *w*/*w* powders in the 500–750 nm wavelength region, in order to check the Li‐PS retention ability of the metal and the metal/metal oxide mixture. The absorption spectra were collected with a V‐570 UV‐vis Spectrophotometer (Jasco Inc.) at room temperature against a DOL:DME 1 : 1 *w*/*w* reference solution.

### Electrochemical Measurements and ex‐situ Investigation

The electrochemical process of S‐SM was studied through CV and EIS employing a VersaSTAT MC Princeton Applied Research (PAR‐AMETEK) multichannel potentiostat. CV was performed within the 1.8–2.8 V *vs*. Li^+^/Li potential range at a scan rate of 0.1 mV s^−1^, while EIS spectra were collected at the OCV condition before cycling and after 1, 5 and 10 CV runs using an alternate voltage signal of 10 mV in the frequency range between 500 kHz and 0.1 Hz. The Li|S‐SM half‐cells were galvanostatically cycled in the 1.7–2.8 V and 1.6–2.8 V voltage ranges, respectively, at constant current rates of either C/3 or 1 C (1 C=1675 mA g_S_^−1^), or by applying current rates increasing every 5 cycles from C/10 to C/8, C/5, C/3, C/2, 1 C, and 2 C, before lowering back to C/10 after 35 cycles, in the 1.7–2.8 V (1 C and 2 C) and 1.8–2.8 V (lower rates) voltage ranges, employing an E/S ratio of 10 μL mg^−1^. For comparison, a Li−S half‐cell using the S control electrode (sulfur loading of 2.65 mg cm^−2^) was galvanostatically cycled between 1.7 and 2.8 V at the constant current rate of C/3 by employing an E/S ratio of 10 μL mg^−1^. An additional Li|S‐SM half‐cell for N/P ratio study was assembled and cycled at a current rate of C/5 in the 1.9–2.8 V voltage range, using an E/S ratio of 15 μL mg^−1^. The Li|SiO_x_‐CM half‐cells were filled with 50 μL of electrolyte using one 18 mm‐diameter monolayer microporous membrane (Celgard 2500) as separator, and studied via galvanostatic cycling between 0.01 and 2.0 V at constant current of 120 mA g^−1^. Li‐ion sulfur full‐cells were assembled by coupling chemically or electrochemically lithiated Li_y_SiO_x_‐CM anodes and S‐SM cathode, separated by an 18 mm‐diameter monolayer microporous membrane (Celgard 2500) soaked with either 15 μL mg_S_^−1^ or 8 μL mg_S_^−1^ (the latter exclusively for high active material loading) of electrolyte. The chemical lithiation of SiO_x_‐CM to achieve the Li_y_SiO_x_‐CM state was performed via direct contact of the pristine electrode surface with a lithium metal foil wet with the electrolyte for 14 or 24 hours under a pressure of 2 kg cm^−2^. Afterwards, the electrode was removed from the lithium foil, dried for 60 minutes under vacuum, and studied in lithium half‐cell and full‐cell using the S‐SM cathode. The electrochemical lithiation to achieve Li_y_SiO_x_‐CM was performed in lithium half‐cell (CR2032 coin‐type cell, MTI Corp.) using the SiO_x_‐CM electrode and 50 μL of electrolyte soaking an 18 mm‐diameter monolayer microporous membrane (Celgard 2500) through either 9 or 5 (the latter exclusively for high active material loading) discharge/charge cycles at constant current of 20 mA g^−1^ in the 0.01–2.0 V voltage range, and a final full discharge held at 0.01 V to achieve the lithiated condition. Then, the Li_y_SiO_x_‐CM electrode was recovered from the above cell in the fully discharged state, dried under vacuum for 60 minutes and employed. Galvanostatic cycling tests were performed on the full‐cells within 0.1 and 2.8 V at current rates of C/5 and C/10, or within 0 and 2.8 V at C/20 (1 C=1675 mA g_S_^−1^). All the galvanostatic cycling tests were carried out by a MACCOR series 4000 battery tests instrument in a chamber set at 30 °C, with maximum fluctuation of ±0.1 °C. *Ex‐situ* SEM was performed on the electrodes with relatively low active material loading, retrieved from the full Li_y_SiO_x_‐CM|S‐SM cells after cycling, using a Zeiss Gemini FESEM 460 relying on a LaB_6_ thermionic gun in secondary electrons mode. Before SEM, the retrieved electrodes were dried under vacuum for 1 hour.

## Supporting Information Summary

Evaluation of polysulfides retention ability of Sn and Sn:MnO_2_ 1 : 1 *w/w* using photographic images (Figure S1). Additional TEM image of the S‐SM powder (Figure S2). Selected voltage profiles of galvanostatic cycling tests on the S‐SM electrode in Li half‐cell at C/3 and 1 C (Figure S3). Voltage profiles and capacity trend of galvanostatic cycling test performed on Li−S control cell at C/3 (Figure S4). Voltage profiles and capacity trend of the galvanostatic test performed on full Li_y_SiO_x_‐CM|S‐SM cell with high active materials loading at various current rates, voltage profiles and capacity trend of corresponding electrochemically lithiation of the Li_y_SiO_x_‐CM electrode, and capacity retention comparison of full‐cells presented in the work (Figure S5).

## Conflict of Interests

The authors declare no conflict of interest.

1

## Supporting information

As a service to our authors and readers, this journal provides supporting information supplied by the authors. Such materials are peer reviewed and may be re‐organized for online delivery, but are not copy‐edited or typeset. Technical support issues arising from supporting information (other than missing files) should be addressed to the authors.

Supporting Information

## Data Availability

The data that support the findings of this study are available from the corresponding author upon reasonable request.

## References

[1] *IEA (2023), Tracking Clean Energy Progress 2023, IEA, Paris, Licence: CC BY 4.0*, **2023**.

[2] A. Manthiram , J. L. Lutkenhaus , Y. Fu , P. Bai , B. G. Kim , S. W. Lee , E. Okonkwo , R. M. Penner , One Earth 2022, 5, 203–206.

[3] A. Manthiram , J. B. Goodenough , Nat. Energy 2021, 6, 323–323.

[4] M. S. Whittingham , Chem. Rev. 2004, 104, 4271–4302. 15669156 10.1021/cr020731c

[5] J. B. Goodenough , K.-S. Park , J. Am. Chem. Soc. 2013, 135, 1167–1176. 23294028 10.1021/ja3091438

[6] A. Manthiram , Nat. Commun. 2020, 11, 1550. 32214093 10.1038/s41467-020-15355-0 PMC7096394

[7] G. Li , S. Wang , Y. Zhang , M. Li , Z. Chen , J. Lu , Adv. Mater. 2018, 30, 1705590. 10.1002/adma.201705590 29577456

[8] A. Manthiram , Y. Fu , S.-H. Chung , C. Zu , Y.-S. Su , Chem. Rev. 2014, 114, 11751–11787. 25026475 10.1021/cr500062v

[9] J. Hassoun , B. Scrosati , Angew. Chem. Int. Ed. 2010, 49, 2371–2374. 10.1002/anie.200907324 20191654

[10] J. Hassoun , B. Scrosati , Adv. Mater. 2010, 22, 5198–5201. 20842661 10.1002/adma.201002584

[11] R. Xu , I. Belharouak , X. Zhang , R. Chamoun , C. Yu , Y. Ren , A. Nie , R. Shahbazian-Yassar , J. Lu , J. C. M. Li , K. Amine , ACS Appl. Mater. Interfaces 2014, 6, 21938–21945. 25425055 10.1021/am504763p

[12] M. Cuisinier , C. Hart , M. Balasubramanian , A. Garsuch , L. F. Nazar , Adv. Energy Mater. 2015, 5, 1401801.

[13] L. Carbone , S. G. Greenbaum , J. Hassoun , Sustain. Energy Fuels 2017, 1, 228–247.

[14] V. Marangon , D. Di Lecce , F. Orsatti , D. J. L. Brett , P. R. Shearing , J. Hassoun , Sustain. Energy Fuels 2020, 4, 2907–2923.

[15] V. Marangon , E. Scaduti , V. F. Vinci , J. Hassoun , ChemElectroChem 2022, 9, e202200374.

[16] V. Marangon , E. Barcaro , L. Minnetti , W. Brehm , F. Bonaccorso , V. Pellegrini , J. Hassoun , Nano Res. 2023, 16, 8433–8447.

[17] S. Li , Z. Luo , L. Li , J. Hu , G. Zou , H. Hou , X. Ji , Energy Storage Mater. 2020, 32, 306–319.

[18] J. Castillo , J. A. Coca-Clemente , J. Rikarte , A. Sáenz de Buruaga , A. Santiago , C. Li , APL Mater. 2023, 11, 010901.

[19] D. Lin , Y. Liu , Y. Cui , Nat. Nanotechnol. 2017, 12, 194–206. 28265117 10.1038/nnano.2017.16

[20] L. Carbone , M. Gobet , J. Peng , M. Devany , B. Scrosati , S. Greenbaum , J. Hassoun , ACS Appl. Mater. Interfaces 2015, 7, 13859–13865. 26057152 10.1021/acsami.5b02160

[21] D. Di Lecce , V. Marangon , H.-G. Jung , Y. Tominaga , S. Greenbaum , J. Hassoun , Green Chem. 2022, 24, 1021–1048.

[22] D. Aurbach , Y. Talyosef , B. Markovsky , E. Markevich , E. Zinigrad , L. Asraf , J. S. Gnanaraj , H.-J. Kim , Electrochim. Acta 2004, 50, 247–254.

[23] A. Bhargav , J. He , A. Gupta , A. Manthiram , Joule 2020, 4, 285–291.

[24] M. Liu , F. Ma , Z. Ge , Z. Zeng , Q. Wu , H. Yan , Y. Wu , S. Lei , Y. Zhu , S. Cheng , J. Xie , Sci. China Chem. 2023, 67, 724–731.

[25] M. Agostini , S. Brutti , J. Hassoun , ACS Appl. Mater. Interfaces 2016, 8, 10850–10857. 27052542 10.1021/acsami.6b01407

[26] C. Hernández-Rentero , V. Marangon , M. Olivares-Marín , V. Gómez-Serrano , Á. Caballero , J. Morales , J. Hassoun , J. Colloid Interface Sci. 2020, 573, 396–408. 32304949 10.1016/j.jcis.2020.03.092

[27] M. Ge , C. Cao , G. M. Biesold , C. D. Sewell , S. Hao , J. Huang , W. Zhang , Y. Lai , Z. Lin , Adv. Mater. 2021, 33, 2004577. 10.1002/adma.202004577 33686697

[28] J. Deng , M. Li , Y. Wang , Green Chem. 2016, 18, 4824–4854.

[29] S.-K. Lee , S.-M. Oh , E. Park , B. Scrosati , J. Hassoun , M.-S. Park , Y.-J. Kim , H. Kim , I. Belharouak , Y.-K. Sun , Nano Lett. 2015, 15, 2863–2868. 25844807 10.1021/nl504460s

[30] C. Shen , M. Ge , A. Zhang , X. Fang , Y. Liu , J. Rong , C. Zhou , Nano Energy 2016, 19, 68–77.

[31] R. Elazari , G. Salitra , G. Gershinsky , A. Garsuch , A. Panchenko , D. Aurbach , Electrochem. Commun. 2012, 14, 21–24.

[32] W. Zhong , Q. Wu , Y. Wu , R. He , C. Liao , S. Cheng , J. Xie , Energy Storage Mater. 2024, 68, 103318.

[33] X. Chen , L. Peng , L. Wang , J. Yang , Z. Hao , J. Xiang , K. Yuan , Y. Huang , B. Shan , L. Yuan , J. Xie , Nat. Commun. 2019, 10, 1021. 30833552 10.1038/s41467-019-08818-6 PMC6399341

[34] S. Tubtimkuna , D. L. Danilov , M. Sawangphruk , P. H. L. Notten , Small Methods 2023, 7, 2300345. 10.1002/smtd.202300345 37231555

[35] E. Barcaro , V. Marangon , M. Mutarelli , J. Hassoun , J. Power Sources 2024, 595, 234059.

[36] J. T. Frith , M. J. Lacey , U. Ulissi , Nat. Commun. 2023, 14, 420. 36702830 10.1038/s41467-023-35933-2 PMC9879955

[37] D. Di Lecce , V. Marangon , W. Du , D. J. L. Brett , P. R. Shearing , J. Hassoun , J. Power Sources 2020, 472, 228424.

[38] A. Benítez , P. Márquez , M. Á. Martín , A. Caballero , ChemSusChem 2021, 14, 3915–3925. 34289246 10.1002/cssc.202101231 PMC8519043

[39] R. Xu , I. Belharouak , X. Zhang , R. Chamoun , C. Yu , Y. Ren , A. Nie , R. Shahbazian-Yassar , J. Lu , J. C. M. Li , K. Amine , ACS Appl. Mater. Interfaces 2014, 6, 21938–21945. 25425055 10.1021/am504763p

[40] J. Xiao , J. Z. Hu , H. Chen , M. Vijayakumar , J. Zheng , H. Pan , E. D. Walter , M. Hu , X. Deng , J. Feng , B. Y. Liaw , M. Gu , Z. D. Deng , D. Lu , S. Xu , C. Wang , J. Liu , Nano Lett. 2015, 15, 3309–3316. 25785550 10.1021/acs.nanolett.5b00521

[41] F. L. Lama , V. Marangon , Á. Caballero , J. Morales , J. Hassoun , ChemSusChem 2023, 16, e202202095. 36562306 10.1002/cssc.202202095

[42] B. Boukamp , Solid State Ionics 1986, 18–19, 136–140.

[43] B. Boukamp , Solid State Ionics 1986, 20, 31–44.

[44] M. R. Busche , P. Adelhelm , H. Sommer , H. Schneider , K. Leitner , J. Janek , J. Power Sources 2014, 259, 289–299.

[45] A. Benítez , Á. Caballero , E. Rodríguez-Castellón , J. Morales , J. Hassoun , ChemistrySelect 2018, 3, 10371–10377.

[46] V. Marangon , C. Hernández-Rentero , M. Olivares-Marín , V. Gómez-Serrano , Á. Caballero , J. Morales , J. Hassoun , ChemSusChem 2021, 14, 3333–3343. 34165920 10.1002/cssc.202101069 PMC8457143

[47] D. Di Lecce , R. Verrelli , J. Hassoun , Green Chem. 2017, 19, 3442–3467.

[48] A. Hadi , J. Zahirifar , J. Karimi-Sabet , A. Dastbaz , Ultrason. Sonochem. 2018, 44, 204–214. 29680604 10.1016/j.ultsonch.2018.02.028

[49] W. Li , Y. Xu , Y. Zhou , W. Ma , S. Wang , Y. Dai , Nanoscale Res. Lett. 2012, 7, 485. 22931369 10.1186/1556-276X-7-485 PMC3499142

[50] P. Verma , P. Maire , P. Novák , Electrochim. Acta 2010, 55, 6332–6341.

[51] W.-S. Chang , C.-M. Park , J.-H. Kim , Y.-U. Kim , G. Jeong , H.-J. Sohn , Energy Environ. Sci. 2012, 5, 6895.

[52] Z. Huang , Z. Deng , Y. Zhong , M. Xu , S. Li , X. Liu , Y. Zhou , K. Huang , Y. Shen , Y. Huang , Carbon Energy 2022, 4, 1107–1132.

[53] Q. Meng , G. Li , J. Yue , Q. Xu , Y.-X. Yin , Y.-G. Guo , ACS Appl. Mater. Interfaces 2019, 11, 32062–32068. 31393103 10.1021/acsami.9b12086

[54] V. Marangon , D. Di Lecce , D. J. L. Brett , P. R. Shearing , J. Hassoun , J. Energy Chem. 2022, 64, 116–128.

[55] A. E. Del Rio Castillo , V. Pellegrini , A. Ansaldo , F. Ricciardella , H. Sun , L. Marasco , J. Buha , Z. Dang , L. Gagliani , E. Lago , N. Curreli , S. Gentiluomo , F. Palazon , M. Prato , R. Oropesa-Nuñez , P. S. Toth , E. Mantero , M. Crugliano , A. Gamucci , A. Tomadin , M. Polini , F. Bonaccorso , Mater. Horiz. 2018, 5, 890–904.

[56] D. Di Lecce , V. Marangon , A. Benítez , Á. Caballero , J. Morales , E. Rodríguez-Castellón , J. Hassoun , J. Power Sources 2019, 412, 575–585.

