# Supplementary material for: Scalable Li‐Ion Battery with Metal/Metal Oxide Sulfur Cathode and Lithiated Silicon Oxide/Carbon Anode
Source: ChemSusChem. 2024 Sep 24;18(1):e202400615. doi: 10.1002/cssc.202400615 (PMC11696217; doi:10.1002/cssc.202400615)
Supplement: Supplementary file 1 — Supporting Information [file CSSC-18-e202400615-s001.pdf]

ChemSusChem

Supporting Information

Scalable Li-Ion Battery with Metal/Metal Oxide Sulfur Cathode and Lithiated Silicon Oxide/Carbon Anode

Edoardo Barcaro, Vittorio Marangon, Dominic Bresser, and Jusef Hassoun*

Figure S1 shows photographic images of vials filled with DOL:DME, 1 mol kg^{-1} LiTFSI, 1 mol kg^{-1} $LiNO_3$, 0.5 wt.% Li_2S_8 solution without any powder addition (reference solution, left-hand side), and with addition of either Sn (central position) or the Sn:MnO_2 1:1 *w/w* mixture (right-hand side), at the initial state (Figure S1a) and after 60 minutes of aging (Figure S1b). The outcomes are used for the study of lithium polysulfides (Li-PS) retention ability (see discussion of Figure 1 in the Manuscript). The images indicate that Sn:MnO_2 1:1 *w/w* can retain the Li-PS much more relevantly than Sn, since the related solution becomes almost transparent after 60 minutes due to the presence of the transition metal oxide (Figure S1b, right-hand side).

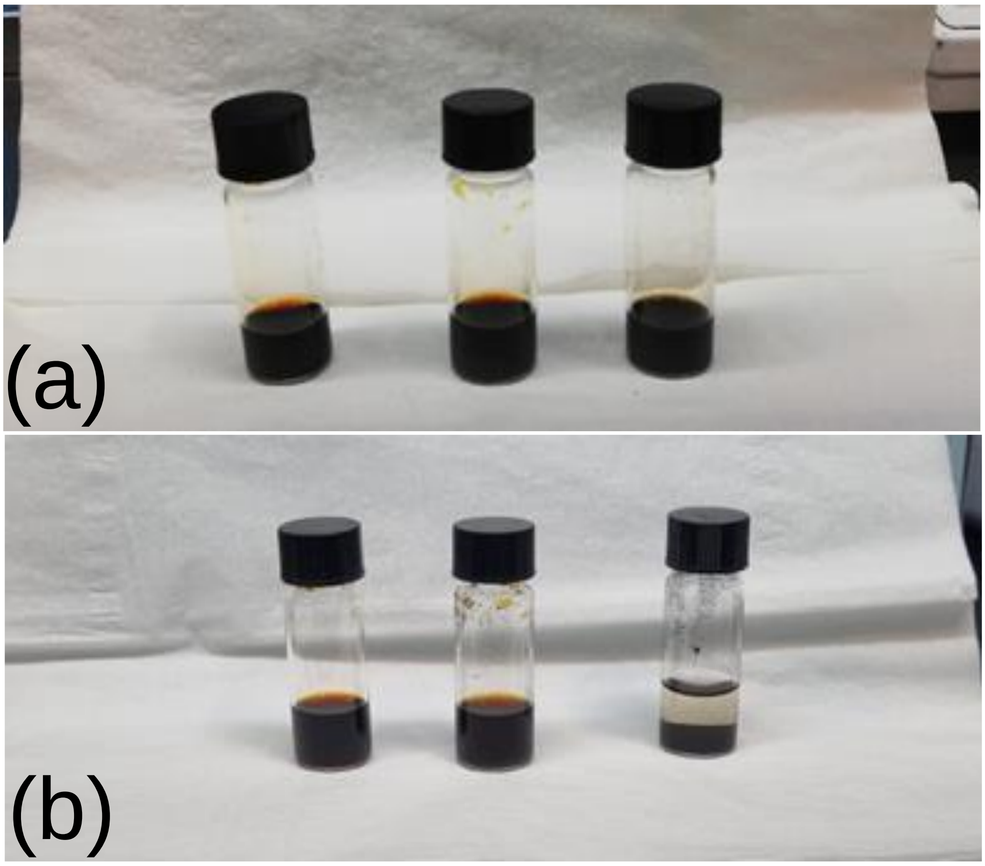

Figure S1. Evaluation of the Li-PS retention ability of Sn and Sn:MnO_2 1:1 *w/w* using photographic images. **(a)** Vials filled with DOL:DME, 1 mol kg^{-1} LiTFSI, 1 mol kg^{-1} $LiNO_3$, 0.5 wt.% Li_2S_8 without any powder addition (reference, left-hand side), and with addition of either Sn (central position) or Sn:MnO_2 1:1 *w/w* (right-hand side), at the pristine state. **(b)** The same solutions after 60 minutes of aging (see Experimental Section in the Manuscript for details, and Figure 1 for further discussion). See Manuscript text for acronyms.

Figure S2 reports an extended-view TEM image of the S-SM powder showing S/MnO_2 domains with a size ranging from some hundred of nm to around 1 μm, and quasi-spherical primary particles of Sn with dimensions smaller than 200 nm. This morphology is considered suitable for actually boosting the Li-S electrochemical process (see Figure 1 in the Manuscript for further discussion).

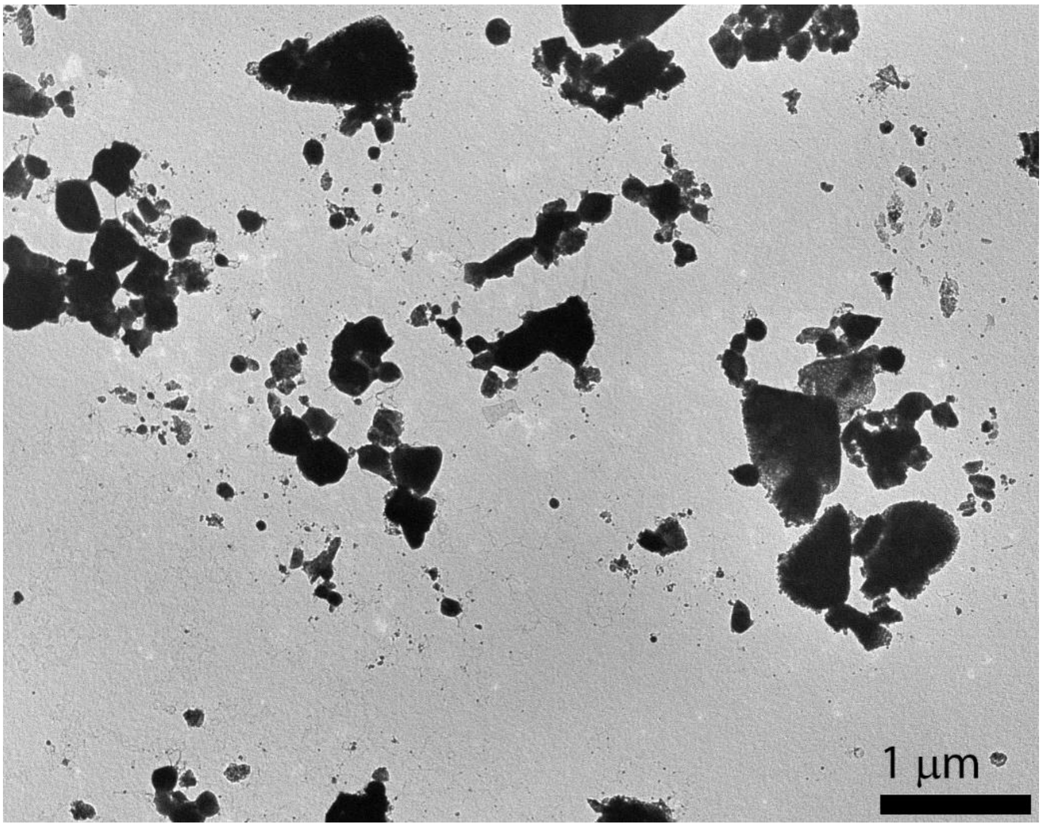

Figure S2. TEM image of S-SM powder additional to the ones reported in Figure 1d and e in the Manuscript. See the Manuscript text for acronyms.

Figure S3 shows voltage profiles selected at the 1st, 50th, 100th, 150th, 200th and 250th cycle of the galvanostatic tests performed on lithium half-cells using the S-SM electrode at a current of C/3 (Figure S3a) and 1C (Figure S3b), corresponding to the cycling trends in Figure 2e and Figure 2f, respectively, in the Manuscript (1C = 1675 mA g_S^{-1}). The aim of the test is to determine the cycle life and the capacity retention of the electrode, and to check the effects of the long-term polarization due to the reaction of the dissolved polysulfides with Li. See Manuscript text for further discussion.

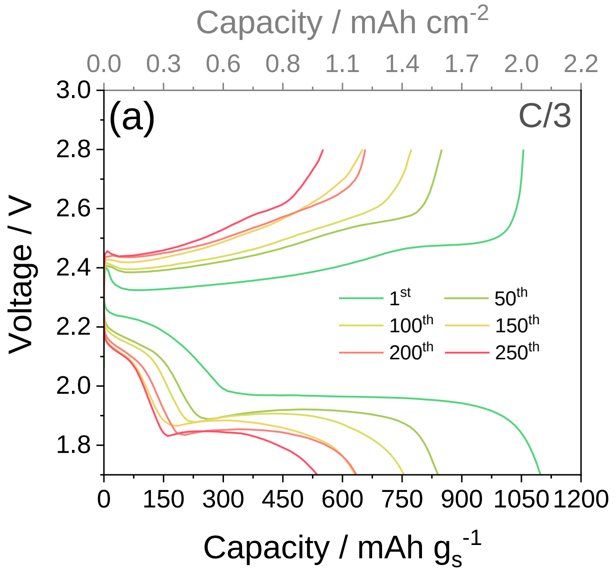

Figure S3. Selected voltage profiles related to galvanostatic cycling tests performed on the S-SM electrode in Li half-cell using the DOL:DME, 1 mol kg^{-1} LiTFSI, 1 mol kg^{-1} $LiNO_3$ electrolyte at the current rates of **(a)** C/3 and **(b)** 1C. Voltage ranges: 1.7-2.8 V for C/3 and 1.6-2.8 V for 1C. Sulfur loading: 1.9 and 2.9 mg cm^{-2}, respectively (electrode geometric area: 1.54 cm^2). E/S ratio: 10 μL mg^{-1}. The corresponding cycling trends are reported in Figure 2 in the Manuscript. Tests performed at 30 °C. See the Manuscript text for acronyms.

Figure S4 shows the voltage profiles (Figure S4a) and capacity trend (Figure S4b) related to a galvanostatic cycling test performed at C/3 on a Li-S cell using the bulk S control electrode. The figure reveals large polarization of the charge/discharge Li-S conversion processes and limited delivered capacity (maximum value of 360 mAh g_S^{-1}), thus evidencing the beneficial effects of Sn and MnO_2 included in the S-SM composite (compare with Figure 2 in the Manuscript).

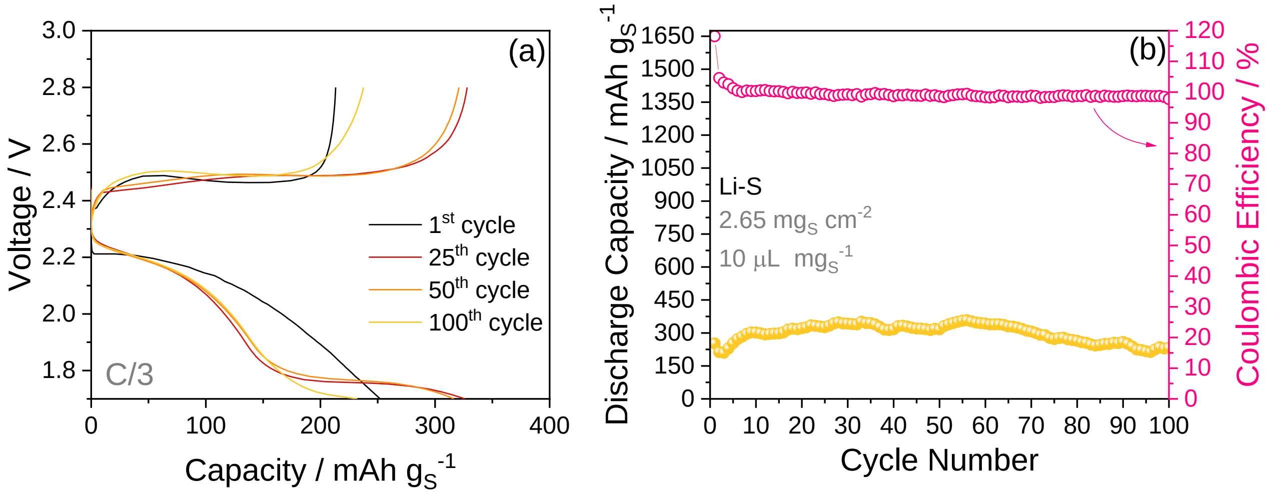

Figure S4. Galvanostatic cycling test of a Li-S half-cell at a current rate of C/3 using a control sulfur electrode in terms of **(a)** selected voltage profile and **(b)** capacity trend (right *y*-axis reports CE); voltage range: 1.7-2.8 V; sulfur loading: 2.65 mg cm^{-2} (electrode geometric area: 1.54 cm^2); E/S ratio: 10 µL mg^{-1}. Test performed at 30 °C.

Figure S5 reports the performance of a full Li_ySiO_x-CM|S-SM cells using an electrochemically lithiated anode and high active material loadings, i.e., 6.30 mg cm^{-2} for the pristine SiO_x-CM and 2.90 mg cm^{-2} for S-SM, as well as an E/S ratio limited to 8 µL mg^{-1} (Figure S5a, b). The electrochemical lithiation of the anode was achieved with a number of charge/discharge cycles limited to 5 followed by one full discharge to achieve the lithiated state (Figure S5c). Despite the challenging conditions, the cycling procedure adopted herein, consisting of the gradual decrease of the current rate, allows an improved capacity retention (Figure S5d) compared to the previous full-cells reported in the Manuscript (compare with Figure 5).

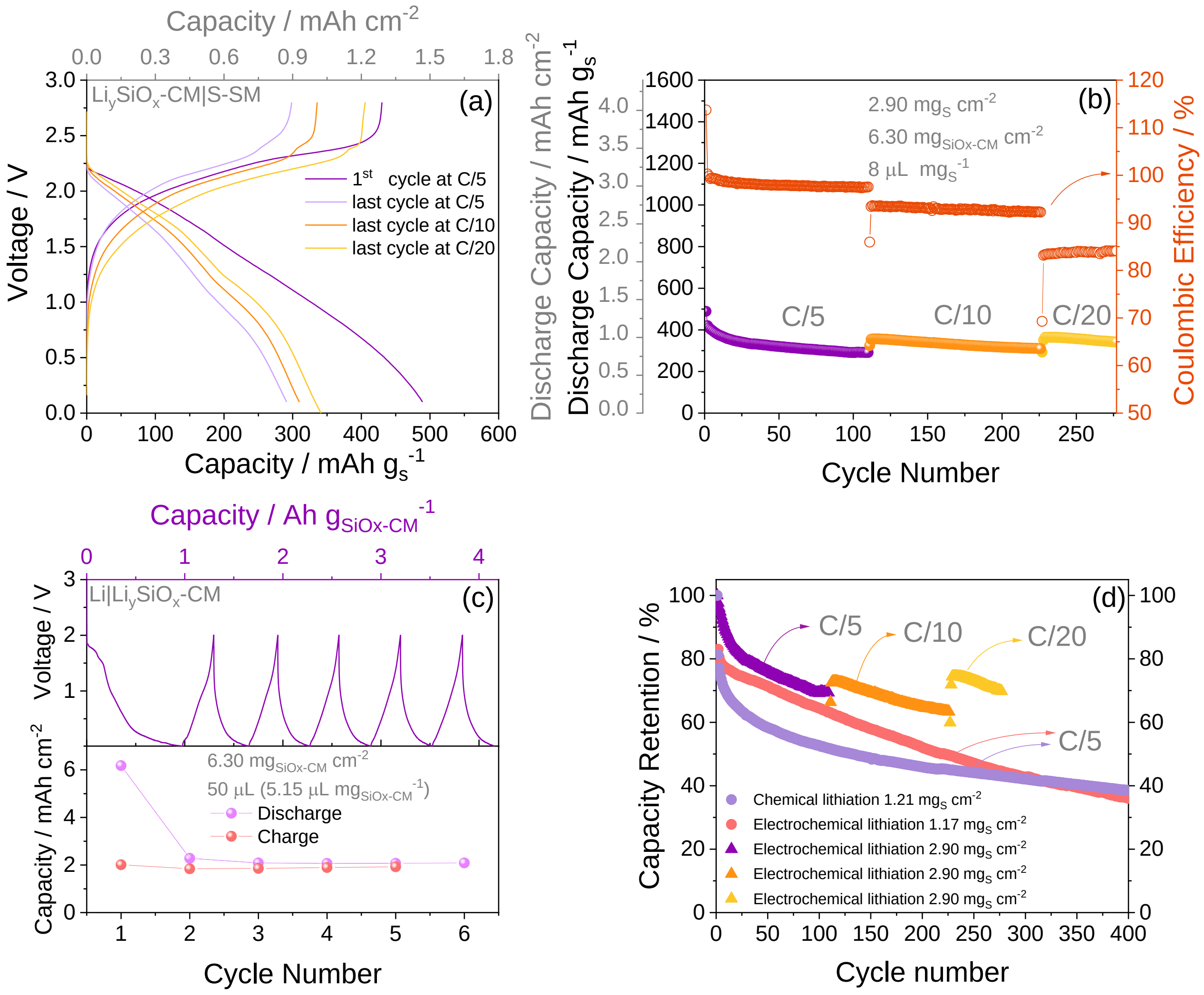

Figure S5. (a, b) Galvanostatic cycling test of a Li_ySiO_x-CM|S-SM battery including an electrochemically lithiated Li_ySiO_x-CM anode with active material loading of 6.30 mg cm^{-2}, a S-SM cathode with sulfur loading of 2.90 mg cm^{-2} and E/S ratio of 8 µL mg^{-1}: **(a)** selected voltage profiles recorded at C/5 (cycles 1-110), C/10 (cycles 111-226), and C/20 (cycles 227-277), voltage ranges: 0.1 – 2.8 V for C/5 and C/10, and 0 – 2.8 V for C/20; **(b)** corresponding cycling trend (right *y*-axis shows CE, additional left *y*-axis displays areal capacity). **(c)** Electrochemical lithiation of a SiO_x-CM electrode (active material loading: 6.30 mg cm^{-2}) in lithium half-cell cycled at 20 mA g^{-1} in the 0.01 – 2.0 V voltage range in terms of voltage profiles (top panel) and capacity trend (bottom panel). **(d)** Comparison in terms of capacity retention plot of the full Li_ySiO_x-CM|S-SM cells presented in the work. Electrodes geometric area: 1.54 cm^2.
